# Design and Applications of MOF-Based SERS Sensors in Agriculture and Biomedicine

**DOI:** 10.3390/s26020499

**Published:** 2026-01-12

**Authors:** Alemayehu Kidanemariam, Sungbo Cho

**Affiliations:** 1 Department of Electronic Engineering, Gachon University, Seongnam-si 13120, Republic of Korea; 2 Department of Semiconductor Engineering, Gachon University, Seongnam-si 13120, Republic of Korea; 3 Gachon Advanced Institute for Health Science & Technology, Gachon University, Incheon 21999, Republic of Korea

**Keywords:** metal–organic frameworks (MOFs), surface-enhanced raman scattering (SERS), agricultural sensors, biomedical sensors, nanomaterial design, pesticide detection, biomarker monitoring, sensor functionalization, sensor stability, portable sensing devices

## Abstract

Metal–organic framework (MOF)-based surface-enhanced Raman scattering (SERS) sensors have emerged as a versatile platform for high-sensitivity and selective detection in agricultural, environmental, and biomedical applications. By integrating plasmonic nanostructures with tunable MOF architectures, these hybrid systems combine ultrahigh signal enhancement with molecular recognition, analyte preconcentration, and controlled hotspot distribution. This review provides a comprehensive overview of the fundamental principles underpinning MOF–SERS performance, including EM and chemical enhancement mechanisms, and highlights strategies for substrate design, such as metal–MOF composites, plasmon-free frameworks, ligand functionalization, and hierarchical or core–shell architectures. We further examine their applications in environmental monitoring, pesticide and contaminant detection, pathogen identification, biomarker analysis, and theranostics, emphasizing real-sample performance, molecular selectivity, and emerging integration with portable Raman devices and AI-assisted data analysis. Despite notable advances, challenges remain in reproducibility, quantitative reliability, matrix interference, scalability, and biocompatibility. Future developments are likely to focus on rational MOF design, sustainable fabrication, intelligent spectral interpretation, and multifunctional integration to enable robust, field-deployable sensors. Overall, MOF-based SERS platforms represent a promising next-generation analytical tool poised to bridge laboratory innovation and practical, real-world applications.

## 1. Introduction

Surface-Enhanced Raman Scattering (SERS) has been recognized as one of the most efficient vibrational spectroscopic methods for trace analysis of chemicals and biological molecules [1,2,3,4]. SERS enhances the inherently weak Raman scattering of molecules by using the strong LSPR signals of nearby metallic nanostructures (Au, Ag, or Cu), enabling highly sensitive, rapid, non-destructive, and label-free molecular fingerprint analysis, even at the single-molecule level [5,6,7,8]. Despite the strong interest in SERS for environmental, food, healthcare, and bioanalytical applications, its use is limited by several challenges, including poor reproducibility, low molecular specificity, and the instability of metallic nanostructures under varying environmental conditions [9,10].

Metal–organic frameworks (MOFs) are a novel kind of porous crystalline materials, which consist of transition-metal ions or clusters linked to organic linkers, have lately gained consideration as effective platforms to overcome the intrinsic limitations of traditional SERS substrates [11,12]. The high surface area, tunable pore morphology, as well as high chemical functionalization of MOFs, provide an efficient platform for selective adsorption, pre-concentration, and orienting target molecules close to plasmonic “hotspots” to simultaneously intensify SERS signals as well as recognize molecules selectively [13,14].

Carbon-based 2D materials, especially those of the graphene family, have recently emerged as highly promising platforms for sensor development due to their tunable physicochemical properties, including high surface area, mechanical robustness, chemical stability, and ease of functionalization [15]. These materials have been successfully applied in field-effect transistor, electrochemical, and Raman-based sensors, offering ultralow detection limits, high specificity, and compatibility with portable or point-of-care devices.

Additionally, MOFs provide an efficient platform to control accurately metallic nodes, organic linkers, as well as topological features, which provide unprecedented opportunities for modulating SERS-active interfaces at nanoscale precision [16,17]. Incorporating plasmonic nanoparticles into or onto MOF lattices through in situ or post-modification methods produces hybrids that combine EM and chemical enhancement, improving SERS sensitivity, stability, and selectivity [18,19,20].

The integration of MOF chemistry and SERS methodology provides novel routes for creating novel sensing systems with potential applications well beyond conventional detection limits [21,22]. In the field of agriculture, SERS-based MOF sensors have received increasing attention as tools for pesticide, veterinary drug, heavy metal, and mycotoxin detection, thus providing a rapid screening tool for assessing food safety and environmental conditions in real-world settings [23,24]. In biomedicine, the potential of SERS-based MOF hybrids for identifying pathogens, biomarkers of disease, therapeutic agents, bioimaging, and theranostics is vast [25,26,27]. A key advantage of MOF hybrids is their tunable physicochemical properties, which enhance their affinity for biomolecules and contaminants, leading to improved detection sensitivity in complex environments [28].

Nonetheless, several scientific and technological issues still need to be addressed to make SERS platforms based on MOFs functional in real-world applications [29]. The most significant problems include the reproducible creation of homogeneous hotspots, optimization of MOF-plasmonic interfaces, as well as implementing hybrid materials into a portable or automated sensing platform [30,31]. Ensuring this requires an in-depth understanding of SERS-active MOF design principles, along with an investigation of structure-property relationships of SERS-active MOFs [32].

This review systematically summarizes recent progress in the design and application of MOF-based SERS sensors, with a particular focus on their roles in agricultural and biomedical analysis. It begins with an overview of SERS fundamentals, including key enhancement mechanisms and the synergistic roles of MOFs and plasmonic materials. The discussion then highlights recent strategies for the preparation of MOF-based SERS substrates, emphasizing approaches for compositional tuning, functionalization, and fabrication methods that establish a foundation for advanced analytical performance. Subsequent sections examine the applications of these hybrid systems in agriculture and biomedicine, including real-sample analyses, to demonstrate the rapid progress and practical potential of MOF–SERS technologies. The review concludes by outlining the existing challenges, emerging opportunities, and future directions for the field particularly the integration of MOF-based SERS platforms with portable Raman devices and artificial intelligence assisted data analysis tools for intelligent, high-throughput sensing applications.

## 2. Fundamentals of MOF-Based SERS Sensors

### 2.1. Fundamentals of Surface-Enhanced Raman Scattering (SERS)

**Scheme 1:**
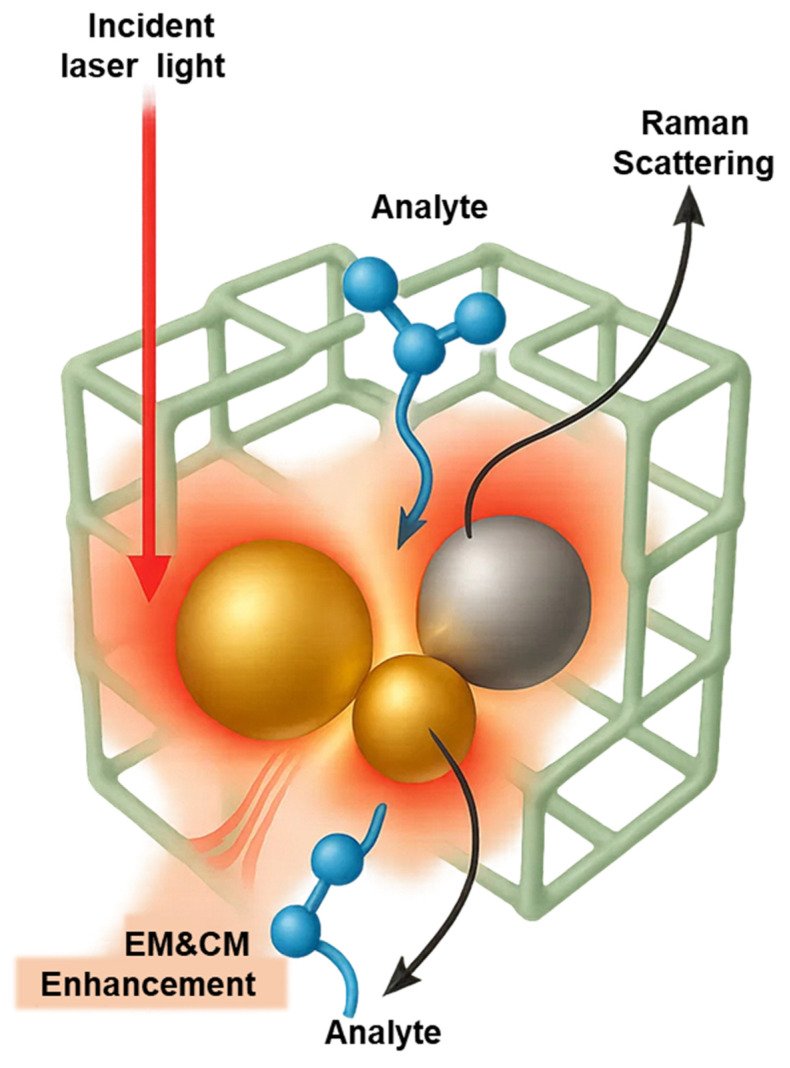
Overview of the EM and CM enhancement mechanisms that govern SERS performance and their integration with MOFs for improved Raman signal amplification and molecular selectivity.

SERS refers to a remarkable optical phenomenon where the Raman signals of molecules become dramatically stronger when they are close to certain nanostructured metal surfaces. This amplification mainly stems from two intertwined mechanisms: EM and chemical (CM) enhancement (Scheme 1) [33,34,35]. In the EM mechanism, incident light excites localized surface plasmon resonances (LSPRs) in noble metal nanostructures most commonly silver, gold, or copper [36]. These collective oscillations of conduction electrons produce intensely confined EM fields at “hotspots,” such as narrow gaps between particles or sharp edges. Molecules situated in these regions experience an enormous local field, often leading to signal boosts of six to eight orders of magnitude, sometimes even under carefully optimized conditions [37]. For MOF-based SERS platforms, excitation wavelengths in the visible range, particularly around 532–633 nm for Ag or Au nanoparticles, have been reported to provide the best signal enhancement, balancing resonance with the plasmonic substrate and analyte absorption.

The CM mechanism, on the other hand, is more subtle. It involves charge-transfer interactions between the analyte and the metal (or semiconductor) surface. When the Fermi level of the substrate aligns favorably with the highest occupied and lowest unoccupied molecular orbitals (HOMO and LUMO) of the analyte, transient charge transfer can occur [38]. This modifies the molecule’s polarizability and thus its Raman scattering cross-section. While this route usually produces more modest enhancement perhaps 10 to 100 times it strongly influences which molecules are most responsive and how their spectra shift [39]. In practice, these two mechanisms often overlap. The challenge has long been to obtain both high EM amplification and good molecular selectivity, something that pure metal nanostructures rarely achieve on their own. This is where MOFs demonstrate their advantages.

The enhancement factor (EF) is a key metric that quantifies the amplification of Raman signals achieved by SERS substrates and provides a comparative measure of sensor performance [40]. In MOF-based SERS platforms, the EF arises from the synergistic interplay of electromagnetic (EM) enhancement at plasmonic hotspots and chemical (CM) enhancement via analyte–MOF interactions. Typical EFs for MOF–metal nanoparticle hybrids range from 10^4^ to 10^8^, depending on the MOF topology, pore environment, plasmonic nanoparticle size, and distribution. In MOF-based SERS systems, EM enhancement is generally the dominant contributor to signal amplification, originating from localized surface plasmon resonances in metallic nanoparticles embedded within or on the MOF. These plasmonic hotspots generate intense local electromagnetic fields, producing major signal boosts. CM enhancement, though smaller in magnitude, arises from charge-transfer interactions between the analyte and the MOF or metal nodes, which can improve selectivity and further enhance Raman scattering. The synergy between EM and CM mechanisms, facilitated by the MOF’s tunable porosity and functional groups, ensures both high sensitivity and molecular specificity in SERS detection.

In practical Raman measurements, the laser intensity and spot size are key factors influencing signal strength [41]. Higher laser intensity and smaller spot size can increase local electromagnetic fields and enhance SERS signals, but may also induce sample heating or photodegradation [42]. These considerations are particularly important when comparing the performance of MOF-based SERS substrates under different experimental conditions, ensuring reproducibility and reliability of measurements.

The spot size (*d*) of the Raman laser on the sample can be estimated using the diffraction-limited formula [43]:

$$d\approx \frac{1.22\;\lambda }{\mathrm{NA}}$$

where λ is the laser wavelength and NA is the numerical aperture of the objective lens. Smaller spot sizes concentrate the laser power into a reduced area, increasing the local field at SERS hotspots and enhancing Raman signals, but careful optimization is needed to prevent thermal damage. These considerations are particularly important when comparing the performance of MOF-based SERS substrates under different experimental conditions, ensuring reproducibility and reliability of measurements.

### 2.2. The Synergy Between MOFs and SERS

MOFs are crystalline, highly porous materials constructed from metal ions or clusters bridged by organic linkers. What makes them so appealing is their tunability everything from the pore size to the surface chemistry can be engineered almost at will [44]. When combined with SERS-active materials, MOFs add several layers of functionality that go beyond what metallic substrates can typically offer.

**Figure 1:**
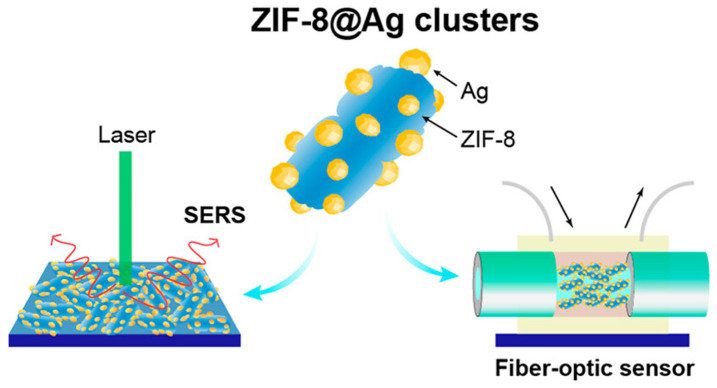
MOF-integrated plasmonic nanostructures enhance molecular enrichment and hotspot stability, enabling high-performance SERS sensing in agriculture and biomedicine. Reproduced with permission ACS [27].

Recently, Tran et al. reported the synergistic interplay between MOFs and plasmonic nanostructures is well exemplified by a ZIF-8@Ag heterostructure engineered for dual SERS and fiber-optic LSPR sensing (Figure 1) [27]. In this system, the porous ZIF-8 framework enhances analyte enrichment and prevents Ag nanoparticle aggregation, while Ag nanoparticles provide strong localized surface plasmon resonances. This cooperative effect produces both electromagnetic (EM) and chemical (CM) enhancement, boosting sensitivity and signal reproducibility, with a SERS enhancement factor of 5.91 × 10^8^ and a detection limit of 0.646 nM for nitrofurantoin. The same architecture also achieves exceptional LSPR performance (detection limit 1.13 × 10^−10^ μM for 4-nitroaniline) (Table 1). Similar designs with Au@ZIF-8 hybrids further illustrate how MOF–plasmonic integration enables analyte preconcentration, selective recognition, and stable plasmonic enhancement [45]. Extending this strategy to magnetic substrates, Fe_3_O_4_@ZIF-67@Ag has been developed for trace detection of malachite green (MG) in water and fish samples [46]. The high surface area of ZIF-67 promotes efficient adsorption of MG, and the combination of EM contribution from Ag nanoparticles with the CM effect of ZIF-67 increases hotspot density, resulting in strong, uniform, and stable SERS signals. The magnetic feature allows easy separation from complex matrices, and the substrate can be regenerated via simple ethanol washing, enhancing cost-effectiveness and practical applicability.

#### 2.2.1. Molecular Enrichment and Preconcentration

Thanks to their well-defined pores and large surface areas, MOFs can trap and preconcentrate analyte molecules on the plasmonic hotspots [47]. For example, UiO-66/AuNP hybrids were used for detecting carcinogenic heterocyclic amines in food and Sudan Red 7B in chili products, achieving recoveries of 82–114% with low RSDs [48]. Moreover, In biomedical applications, MOF-derived porous Fe_3_O_4_ nanorods (MOF-IONRs) capture extracellular vesicles carrying HGSOC-specific protein biomarkers, enabling multiplexed SERS detection with a limit of 2.13 EVs/µL and %RSD < 10% [49]. The study highlights how MOF-based platforms can enhance molecular enrichment and preconcentration, demonstrating the potential of MOF–SERS hybrids for highly sensitive, multiplexed, and clinically relevant biomarker detection in complex biological matrices.

Xu et al. encapsulated PVP-capped Au nanoflowers within a ZIF-67 shell (Au NFs@ZIF-67), where the branched Au core generated intense EM hotspots while the ZIF-67 shell selectively enriched histamine molecules and stabilized the plasmonic interface [50]. The use of 4-mercaptobenzoic acid (4-MBA) as an internal Raman standard effectively minimized signal fluctuation, enabling quantitative detection down to 8.7 × 10^−8^ M and successful monitoring of fish spoilage. This core–shell architecture demonstrates how MOF encapsulation enhances sensitivity and reproducibility, although precise control over shell thickness is required to avoid signal attenuation.

Similarly, MOF-on-MOF heterostructures further enhance sensitivity through hierarchical molecular trapping. A Zn-BTEC@ZIF-8 architecture was used to adsorb tetracycline molecules via hydrogen bonding and π − π interactions, leading to local aggregation and fluorescence amplification. Upon exposure to histamine, structural modulation disrupted the stabilized framework, producing signal changes that enabled highly sensitive detection in environmental samples [51]. This illustrates how layered MOF structures can synergistically preconcentrate analytes and facilitate efficient signal transduction.

Integrating plasmonic nanoparticles with MOFs provides another level of synergy. A high-performance SERS sensor was constructed by combining Au nanoparticles with ZIF-8 photonic crystals (ZIF-8 PCs), where the porous MOF matrix promoted analyte enrichment while structural engineering optimized plasmonic hotspot distribution [52]. This cooperative design significantly amplified electromagnetic (EM) fields at the hotspots, achieving ultra-sensitive, reproducible detection of diverse analytes including 4-mercaptobenzoic acid, Rhodamine 6G, methylene blue, Thiabendazole, and Parathion-methyl without complex pretreatment. Molecular dynamics simulations confirmed the self-enrichment effect, highlighting the critical role of MOF–plasmonic synergy in enabling sensitive, selective, and practical on-site SERS analysis.

MOF-coated plasmonic paper substrates further highlight the balance between simplicity and selectivity. An Au@ZIF-8 SERS paper platform enabled efficient capture of volatile biogenic amines through MOF porosity, while surface functionalization with 4-MBA improved selectivity via aldehyde–amine interactions [53]. This design allowed ppb-level detection of spoilage-related amines in real samples, emphasizing portability and practicality, though at the expense of the ultralow detection limits achievable with nanostructured plasmonic cores.

**Figure 2:**
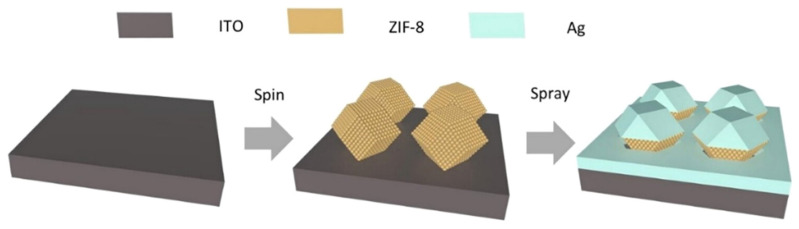
Schematic illustration of the fabrication of an Ag/ZIF-8 SERS substrate, showing ZIF-8 nanocrystal deposition followed by Ag layer coating to form plasmonic nanogaps. Reproduced with permission ACS [54].

The general applicability of ZIF-8 as an enrichment layer is further supported by Ag/ZIF-8 substrates in which porous ZIF-8 nanocrystals were used to localize analyte molecules within engineered plasmonic nanogaps (~10 nm), producing strong near-field electromagnetic coupling and highly reproducible SERS signals [54]. Although demonstrated using 4-aminothiophenol as a model analyte, this architecture provides a transferable design principle for histamine sensing, where efficient molecular confinement near hotspots is critical for overcoming weak intrinsic metal–amine interactions (Figure 2). The study highlights a key trade-off: precise nanogap and MOF thickness control can yield exceptional sensitivity and reproducibility, but requires increased fabrication complexity compared to simpler MOF-coated plasmonic substrates.

#### 2.2.2. Selective Molecular Recognition

Unlike bare metal nanoparticles, MOFs can be chemically selective. Functional groups on their linkers or ones introduced later through post-synthetic modification can interact with target molecules via hydrogen bonding, π − π stacking, or coordination chemistry. This targeted affinity reduces background interference and makes spectral identification more reliable, especially in complex mixtures [55].

Recently, Xue et al. reported a selective MOF-based SERS substrate was developed by integrating Ag nanoparticles with UiO-66(NH_2_) coated in a polydopamine layer, followed by molecular imprinting to generate Ag@UiO-66(NH_2_)/PDA-MIPs [32]. The imprinted cavities provided specific binding sites for the target dye, enabling strong molecular recognition and reducing interference from non-target species. This selective enrichment led to a clear linear SERS response at 1596 cm^−1^ across 10^−8^–10^−10^ mol L^−1^, achieving an ultralow detection limit below 10^−10^ mol L^−1^ with excellent correlation (R^2^ = 0.9985). The substrate also performed effectively in real river-water samples, demonstrating the role of MOF-assisted imprinting in enhancing selective molecular detection.

Selective detection of tetracyclines is critical due to their widespread environmental impact. A Zn-based MOF (HNU-55) was employed to construct a ratiometric fluorescence sensor, with the MOF serving as the detection signal and rhodamine B (RhB) as an internal reference [56]. The sensor selectively recognizes tetracyclines through chelation between Zn^2+^ sites and hydroxyl groups of the analytes, while π − π stacking interactions and intermolecular hydrogen bonding further enhance analyte binding and restrict conformational rotation, resulting in amplified fluorescence signals. This design enabled precise differentiation of four tetracyclines, with detection limits of 4.6 nmol/L for doxycycline, 2.6 nmol/L for tetracycline, 4.7 nmol/L for oxytetracycline, and 7.5 nmol/L for chlortetracycline. Application of the sensor in lake water samples achieved satisfactory recoveries, demonstrating robust real-sample performance. Additionally, a portable optosmart sensing system was developed for on-site visual quantification, highlighting the practical potential of MOF-enabled selective recognition strategies in environmental monitoring.

Castration-resistant prostate cancer (CRPC) necessitates early and accurate detection of therapeutic drugs and biomarkers, yet the complex and diverse structures of these drugs make selective sensing challenging. Lanthanide MOF (Ln-MOFs) have emerged as effective platforms for molecular recognition due to their intense luminescence, tunable chemical environment, and highly conjugated linkers, such as H_2_L (5-(4-(triazole-1-yl)phenyl)isophthalic acid). By constructing bimetallic EuxTb_1−x_-MOFs, researchers have developed luminescent sensor arrays capable of discriminating CRPC drugs—even in mixtures—using multivariate analysis techniques like principal component analysis (PCA) and hierarchical cluster analysis (HCA) [57]. Specifically, Eu_0.096_Tb_0.904_-MOF (MOF 3) exhibits strong emission at 543 and 614 nm and, when combined with hydroxyflutamide to form the composite MOF 3@hydroxyflutamide, enables highly selective detection of CRPC biomarkers such as the androgen receptor (AR) and prostate-specific antigen (PSA) in real serum samples. Mechanistic studies including luminescence lifetime measurements, zeta potential analysis, and density functional theory (DFT) calculations confirm that the MOF’s structural and chemical features mediate selective molecular recognition, demonstrating how MOFs can be rationally engineered to enhance analyte specificity in complex biological matrices.

#### 2.2.3. Controlled Hotspot Distribution

Embedding or decorating metallic nanoparticles within the MOF matrix allows better spatial control of hotspots. The framework keeps the particles from clumping together, ensuring that the enhanced regions remain stable and reproducible over time [58].

Random aggregation and uncontrolled nanogaps between plasmonic nanoparticles often limit the reproducibility and quantitative reliability of conventional SERS measurements. To overcome this, single core–shell plasmonic nanoparticles have been developed, in which a silver nanoflower (AgNF) core is encapsulated by a porous ZIF-8 shell [59]. The inherent nanostructure of the AgNF core provides well-defined nanogaps that serve as stable plasmonic hotspots, while the ZIF-8 shell prevents nanoparticle aggregation and maintains consistent hotspot distribution. The porous ZIF-8 layer also facilitates molecular enrichment by capturing target analytes, positioning them directly within the vicinity of the plasmonic hotspots for enhanced Raman response. This design results in highly stable and reproducible SERS signals, with reported relative standard deviations (RSDs) as low as 2.29% for single nanoparticles and 9.86% for multiple particles. Moreover, the controlled hotspot architecture of these core–shell MOF–plasmonic hybrids allows integration with advanced data processing techniques. For instance, coupling with deep learning algorithms enables accurate identification of volatile organic compounds (VOCs) such as gaseous glutaraldehyde, achieving classification accuracies up to 97.5%.

To achieve high sensitivity in SERS, the spatial distribution of plasmonic “hotspots” and the local concentration of analyte molecules are critical factors. A recent approach involves constructing SERS substrates using gold nanostars (Au NSs) encapsulated within a nickel-cobalt layered double hydroxide (LDH) shell, synthesized via a zeolitic imidazolate framework (ZIF) sacrificial template and subsequent etching with nickel ions [60]. In this architecture, the LDH shell serves dual purposes: it acts as an adsorption medium that preconcentrates target molecules near the plasmonic surface and simultaneously prevents aggregation of the Au NSs, ensuring stability and uniformity of the hotspots. The porous shell facilitates close proximity of analytes to the metallic core, enhancing EM interactions, which was confirmed through UV–Vis absorption experiments and density functional theory simulations. This design enabled the detection of Rhodamine 6G at concentrations as low as 10^−9^ M with a relative standard deviation below 10%, demonstrating that introducing a well-engineered adsorption layer around plasmonic nanostructures is an effective strategy to control hotspot distribution and improve SERS signal reproducibility.

Moreover, Au–Ag alloy nanourchins were integrated with MOFs@Au to construct a SERS substrate for the highly sensitive detection of oxytetracycline (OTC) in food samples [61]. The layered MOFs provided a high surface area scaffold that immobilized a substantial number of Au nanoparticles, ensuring a uniform and stable distribution of plasmonic hotspots. The Au–Ag nanourchins, functionalized with toluidine blue (TB) as SERS signal tags, introduced abundant sharp tips, which further amplified the local EM field. In the absence of OTC, minimal interaction occurred between the TB-labeled nanourchins and the MOF@Au substrate, resulting in negligible Raman signals. Upon introduction of OTC, an enzyme-free dual-cycle amplification strategy, driven by entropy-mediated DNA reactions and catalytic hairpin assembly (CHA), facilitated the association of nanourchins with the MOF@Au, leading to a synergistic enhancement of the TB Raman signal. This precise spatial arrangement of plasmonic nanoparticles enabled exceptionally strong SERS enhancement, yielding a detection limit as low as 6.97 × 10^−15^ mol/L for OTC. These results demonstrate how careful control of nanoparticle distribution within MOFs can significantly improve hotspot uniformity, signal reproducibility, and overall sensor performance.

#### 2.2.4. Charge-Transfer Pathways

Some MOFs, particularly those with semiconductive or conjugated backbones, can also contribute to charge-transfer-based enhancement [62]. This means the MOF itself may participate in the SERS process, offering a secondary route for signal amplification even without traditional plasmonic effects. Together, these properties give rise to MOF–SERS hybrids that combine the sensitivity of plasmonic nanostructures with the selectivity and chemical richness of MOFs a promising mix for sensors meant to operate outside the lab, in real-world conditions [63].

SERS has become a highly sensitive technique for detecting a wide range of molecules, including volatile gaseous analytes. Developing stable, efficient, and reusable SERS substrates for gas-phase detection remains a challenge due to weak analyte substrate interactions and potential nanoparticle aggregation. Zhang et al. reported a novel Cu-Ag@ZIF-8 film, consisting of a silver nanoparticle (AgNP) core coated with a porous ZIF-8 shell on copper foil [64]. The ZIF-8 shell not only stabilizes the AgNPs but also facilitates adsorption and orientation of gaseous molecules near the plasmonic hotspots, enabling charge-transfer interactions that enhance the Raman scattering signal. This dual contribution of EM amplification from the Ag core and chemical enhancement from the MOF shell allows sensitive detection of p-aminothiophenol (PATP) and 4-mercaptophenol (4-MP) with detection limits as low as 9.12 × 10^−10^ M and 9.91 × 10^−9^ M, respectively. Furthermore, the substrate has been applied to detect benzaldehyde vapors emitted from commercial oil paints. By integrating deep learning algorithms for spectral analysis, accurate identification of benzaldehyde in complex VOC mixtures was achieved with 97.7% accuracy. This example demonstrates how MOF-assisted charge-transfer pathways, in combination with plasmonic enhancement, can significantly improve the sensitivity and selectivity of SERS detection for gaseous molecules.

SERS offers a powerful approach for detecting gaseous small molecules, yet its performance is often limited by the low adsorption of analytes on conventional plasmonic surfaces and their inherently weak Raman scattering cross-sections. To overcome these challenges, Peng et al. reported a nucleophilic addition strategy has been developed using a core–shell Ag@Au nanoparticle system functionalized with 4-nitrophenylhydrazine (4-NPH) and encapsulated within a ZIF-8 porous framework [65]. In this configuration, the ZIF-8 scaffold provides abundant adsorption sites, enhancing analyte localization near plasmonic hotspots, while the 4-NPH acts as a Raman reporter that participates in a charge-transfer reaction with acetone, modulating its polarizability and resulting in amplified Raman signals. Density functional theory (DFT) studies reveal how the reaction alters the Raman spectral features of 4-NPH, offering a molecular-level understanding of the selective detection mechanism. The combination of enhanced analyte adsorption and the charge-transfer interaction between the reaction product and the metal surface significantly improves SERS sensitivity and reproducibility. As a result, the platform achieves low relative standard deviations (RSD~4.8%) and can detect acetone at concentrations as low as 100 ppb, demonstrating both high sensitivity and reliability. This approach exemplifies how MOFs can facilitate charge-transfer pathways to augment SERS signals, particularly for molecules that are otherwise challenging to detect due to weak Raman activity or low surface affinity.

A carbon dots (CDs)-decorated, MOF-derived porous Co_3_O_4_ (CDs–Co_3_O_4_) sensor was developed to exploit charge-transfer mechanisms for enhanced humidity detection [66]. The incorporation of CDs provides additional charge-transfer sites within the MOF-derived matrix, while the porous Co_3_O_4_ framework facilitates efficient interaction with water molecules. Fabricated as a miniaturized microwave resonator (0.98 × 0.80 × 0.22 mm^3^) using integrated passive device technology, the sensor exhibits high stability, reproducibility, and suitability for wearable applications. Under microwave excitation, the CDs–Co_3_O_4_ system demonstrates enhanced sensitivity, with a frequency shift of 3.40 MHz/% RH and a loss variation of 0.15 dB/% RH across 5–99% relative humidity, representing 49.7% and 20.5% improvements over Co_3_O_4_ sensors without CDs. This enhancement arises from synergistic charge-transfer interactions between water molecules and the MOF-derived framework, coupled with microwave interactions, analogous to the chemical enhancement in MOF-based SERS. The sensor also shows high selectivity against other volatile compounds and can monitor real-time respiratory patterns and subtle humidity changes, illustrating the practical utility of MOF-derived charge-transfer pathways for sensitive and reliable detection in real-world applications.

### 2.3. Design Principles for MOF-Based SERS Substrates

The effectiveness of a MOF-based SERS substrate depends largely on how well the MOF and plasmonic components work together [67]. Designing such systems usually revolves around three aspects: the framework composition, structural architecture, and surface chemistry.

#### 2.3.1. Metal Nodes and Linkers

The choice of metal centers such as Cu^2+^, Zr^4+^, or Fe^3+^ dictates the framework’s stability, conductivity, and potential interactions with analytes [68]. Likewise, the organic linkers (terephthalates, imidazolates, porphyrinates, and so on) control the pore geometry and chemical environment [69]. For instance, electron-rich linkers may promote charge transfer, while ligands bearing –NH_2_, –COOH, or –SH groups can selectively bind particular molecules [70].

The choice and modification of metal centers in MOF-derived substrates play a critical role in determining the chemical reactivity, structural stability, and sensing performance of the final material. In one study, a cerium-based MOF (Ce-MOF) was used as a precursor to synthesize a Ni-doped CeO_2_ nanostructure via a hydrothermal encapsulation and calcination process [71]. Introducing Ni into the CeO_2_ framework effectively tuned the electronic properties and created additional active sites, analogous to tailoring metal nodes in MOFs for enhanced analyte interactions. Physical characterization revealed that Ni doping led to a porous, hollow spherical morphology with abundant reactive oxygen sites, which significantly improved surface reactivity. Functionally, the Ni-CeO_2_ composite exhibited remarkable H_2_S detection performance, achieving a low detection limit of 8.68 ppb, excellent linearity (R^2^ = 0.998), and a high sensitivity response (Ra/Rg = 108 at 100 °C). The improved performance was attributed to the synergistic effect of Ni incorporation and the CeO_2_ framework, which enhanced oxygen vacancy formation and electron transfer pathways, demonstrating how careful selection and modification of metal nodes can optimize MOF-derived sensing substrates.

#### 2.3.2. Incorporation of Plasmonic Nanostructures

Several strategies can be applied in this context. Nanoparticles can be grown inside the MOF (in situ), attached after synthesis (ex situ), or enclosed in core–shell configurations such as Au@MOF or MOF@Ag [72]. Each has trade-offs: core–shell systems often deliver strong EM enhancement and molecular sieving, while embedded designs provide stability and protect the plasmonic component from degradation.

In a representative example of plasmonic integration, a MOF-199/Ag@Au hybrid SERS sensor was developed for the highly sensitive detection of dopamine (DA), a key biomarker for neurological diseases, in serum [73]. The copper-based MOF (MOF-199) was synthesized in situ on a copper substrate, providing a stable and porous scaffold for subsequent deposition of silver nanoparticles (Ag NPs). To further enhance plasmonic activity and signal reproducibility, gold nanoshells were grown around the Ag cores through an in situ chemical deposition method, forming a robust core–shell architecture. This synthesis approach is simple, controllable, and cost-effective, while ensuring uniform distribution of plasmonic hotspots. The resulting MOF–plasmonic structure effectively combines EM and chemical enhancement mechanisms, leading to exceptional SERS performance. Using an Azo reaction-based detection method, the sensor achieved an ultralow detection limit of 1 pM DA, representing a two- to four-fold improvement over previous unlabeled SERS approaches. Moreover, a strong linear correlation (R^2^ = 0.976) was observed between the SERS signal intensity at 1140 cm^−1^ and DA concentration over the range 1 pM–0.001 M, demonstrating both qualitative and quantitative detection capabilities. This example highlights how strategic incorporation of plasmonic nanoparticles within MOF matrices can stabilize metallic nanostructures, control hotspot distribution, and significantly enhance SERS sensitivity and reproducibility.

Moreover, Feng et al. reported a procedure to employe an in situ approach, gold nanoparticles (AuNPs) were synthesized within the acid-etched MIL-101(Cr) framework, yielding AuNP/AE-MIL-101(Cr) nanocomposites as an efficient SERS substrate [74]. The MOF scaffold provides a confined environment that uniformly distributes and stabilizes the AuNPs, preventing aggregation and ensuring the formation of abundant and reproducible SERS hotspots. The SERS performance of these nanocomposites was systematically evaluated using model analytes including 4-mercaptophenylboronic acid (4-MPBA), 4-mercaptobenzoic acid (4-MBA), benzidine, and rhodamine 6G (R6G). The results demonstrated excellent stability and low background interference, with benzidine serving as a Raman reporter achieving a limit of detection as low as 6.7 × 10^−13^ mol·L^−1^ and intra- and inter-batch relative standard deviations below 5.2%. This design strategy was further extended to a practical biomedical application: the quantitative detection of human carboxylesterase 1 (hCE1) in human serum. Using AuNP/AE-MIL-101(Cr) as the SERS-active substrate and ELISA-derived colorimetric markers as reporters, hCE1 was detected directly in clinical samples without complex pretreatment. Within the concentration range of 0.1–120 ng·mL^−1^, the SERS signal intensity at 1609 cm^−1^ decreased proportionally with hCE1 concentration (R^2^ = 0.9948), achieving recoveries between 84 and 108% with relative standard deviations below 7.7%.

To enhance SERS detection of Crystal Violet (CV), a MOF(Zr)@Ag nanoparticle substrate was fabricated using a two-step process that strategically integrates plasmonic nanoparticles within the MOF [75]. The Zr-based MOF provides a high specific surface area (~1200 m^2^/g) and abundant porosity, facilitating the formation of plasmonic “hotspots” through the localized surface plasmon resonance (LSPR) of Ag nanoparticles, while simultaneously promoting analyte preconcentration. Organic ligands containing amino (–NH_2_) and sulfhydryl (–SH) groups were incorporated into the MOF structure to enable selective molecular interactions, and benzoic acid was added during synthesis to reduce the coordination number of Zr nodes, creating defect sites that further enhance adsorption of target molecules. This rational design yielded a substrate capable of detecting CV, Rhodamine 6 G (R6G), and 4-Mercaptobenzoic Acid (4-MBA) at ultralow concentrations, with CV residues detectable at levels exceeding parts per billion (ppb) in shrimp tissue samples. The integration of Ag nanoparticles within the MOF matrix ensured spatially controlled hotspots and improved reproducibility, demonstrating the effectiveness of MOF–plasmonic hybrid design for ultrasensitive and stable SERS detection.

A representative example of plasmonic–MOF integration is the one-step synthesis of silver-based metal–organic skeletons (AgMOFs) directly grown on flexible cotton swabs, which act both as a structural scaffold and as a stabilizing substrate during in situ nanoparticle formation [76]. Using benzene-1,3,5-tricarboxylic acid (H_3_BTC) and AgNO_3_ in an ethanol–water mixture, AgMOF crystals rapidly nucleate at the solvent interface and uniformly coat the fibrous cotton network, creating a mechanically robust and hierarchically porous SERS-active architecture. The resulting CS–AgMOF hybrid demonstrates strong plasmonic enhancement due to well-dispersed Ag sites while the MOF prevents nanoparticle aggregation and ensures stable hotspot distribution. When applied as a flexible swab-type SERS substrate, the platform enables sensitive detection of explosives such as RDX and TNT, achieving limits of detection of 0.1 mM and 0.5 mM, respectively. Moreover, integration with a smartphone-based quantitative Raman analysis app highlights the practicality of such MOF–plasmonic hybrids for low-cost, portable sensing.

#### 2.3.3. Morphology and Dimensionality

The external shape and dimensionality of the MOF whether it forms thin nanosheets, nanorods, or hierarchical 3D assemblies affect how analytes diffuse and how light interacts with the material [77]. For example, 2D ultrathin MOFs shorten diffusion paths and expose more active surface, while porous 3D networks enhance multiple scattering and analyte trapping.

A 3D hierarchical SERS substrate was fabricated by assembling Ag nanoparticles onto ZnO nanoparticles derived from porous ZIF-8 frameworks, providing a facile and cost-effective platform for trace detection of multiple analytes, including rhodamine 6G (R6G), crystal violet (CV), tetracycline, and thiram [78]. The hierarchical porous structure and large surface area of the ZIF-8–derived ZnO promote extensive “hotspot” formation and enhanced analyte adsorption, resulting in ultra-sensitive detection with a lowest detectable concentration of 10^−13^ M for R6G and an enhancement factor of 1.8 × 10^8^. The 3D architecture also supports photocatalytic degradation of probe molecules under short UV exposure (<30 min), enabling excellent substrate reusability. Finite-difference time-domain (FDTD) simulations confirmed that the widespread distribution of EM hotspots throughout the hierarchical structure is the primary contributor to the observed SERS enhancement, highlighting the critical role of morphology and dimensionality in optimizing MOF-derived SERS substrates.

The morphology and dimensionality of MOF-based SERS substrates play a crucial role in determining analyte diffusion, adsorption efficiency, and light–matter interactions. Recently, Chen et al. reported fabrication of a MIL-101(Fe)@Ag composite via a photoreduction method to produce a well-defined hybrid nanostructure [79]. The three-dimensional porous framework of MIL-101(Fe) provides abundant adsorption sites, enabling efficient preconcentration of target molecules near the plasmonic silver nanoparticles. The embedded Ag NPs exploit localized surface plasmon resonance effects, while the hierarchical porous morphology of the MOF facilitates uniform nanoparticle distribution and multiple light-scattering pathways. Characterization of the composite using FTIR, UV–Vis spectroscopy, and SEM confirmed the successful formation of the hybrid structure with uniform morphology. SERS testing with PATP revealed a detection limit of 10^−8^ M and a relative standard deviation of 5.3%, demonstrating both high sensitivity and reproducibility. Furthermore, the substrate was successfully applied for formaldehyde detection, achieving a detection limit of 10^−9^ M, with quantitative analysis performed via principal component analysis. This example illustrates how careful control over MOF morphology and dimensionality enhances molecular accessibility, hotspot distribution, and overall SERS performance, highlighting the importance of structural design in developing high-performance MOF–plasmonic substrates.

In another study, a novel SERS substrate was developed by engineering the morphology and dimensionality of a MOF-based system to enable real-time detection of Rhodamine 6G [80]. ZIF-67 was first grown in situ on a polypropylene membrane, forming a uniform and porous layer that provides an accessible surface for analyte adsorption. Subsequently, pre-synthesized Ag nanoparticles were immobilized onto the ZIF-67-coated membrane through electrostatic interactions and covalent Ag–S bonding. The combination of a well-defined MOF morphology and nanoparticle decoration facilitated efficient analyte enrichment near plasmonic hotspots. Under optimized flowing conditions, this substrate enabled rapid SERS detection of Rhodamine 6G within 15 min, exhibiting a relative standard deviation of 10.67% and a linear correlation between analyte concentration and SERS intensity over a broad range (10–3 to 10 μmol/L). This example highlights how careful control of MOF morphology and dimensionality, along with spatially organized plasmonic nanostructures, can significantly improve analyte accessibility, signal reproducibility, and overall SERS performance.

For the first time, a highly sensitive flexible sensor was developed for potentiometric monitoring of Ni^2+^ ions, incorporating 2D Ni-MOF nanosheets as the electroactive material [81]. The sensor was fabricated using a highly porous activated carbon cloth decorated with nitrogen-doped spherical porous carbon nanoparticles derived from low-cost cotton, combined with polypyrrole nanoparticles via a simple carbonization-activation process and subsequent dip-coating in a membrane cocktail containing the Ni-MOF nanosheets. The 2D morphology of the Ni-MOF and the hierarchical porosity of the composite facilitated rapid diffusion and efficient access of Ni^2+^ ions to electroactive sites, resulting in a low detection limit of 2.7 × 10^−6^ M over a wide pH range of 2–8. The sensor also exhibited stable, rapid, and reproducible responses in biological fluids, including saliva and sweat, as well as in tap water, without requiring preconditioning. Additionally, the flexible architecture demonstrated antibacterial activity against Gram-positive (Staphylococcus aureus) and Gram-negative (*Escherichia coli*) bacteria, ensuring safe handling for wearable applications. This design highlights how morphology and dimensionality of MOFs can be strategically leveraged to enhance diffusion, surface accessibility, and overall sensor performance in flexible and wearable platforms.

#### 2.3.4. Surface Functionalization and Post-Synthetic Tuning

Post-synthetic modification (PSM) makes it possible to introduce recognition sites, catalytic moieties, or hydrophilic–hydrophobic balance after the MOF is assembled [82]. Functionalization with aptamers, peptides, or polymers can further extend the range of targets useful, for instance, when dealing with biological fluids or agricultural samples.

Recently, a layered SERS substrate composed of filter paper (FP), silver nanoparticles (AgNPs), and a zeolitic imidazolate framework (ZIF-8) film (FP/Ag/ZIF-8) was engineered to investigate how post-synthetic tuning of MOF layers influences analyte adsorption and SERS performance [83]. By adjusting the thickness of the ZIF-8 coating, it was found that a relatively thick MOF layer (125 nm) provided optimal adsorption of the pesticide probe 4-aminothiophenol (4-ATP), yielding a 1000-fold improvement in detection limit compared with FP/Ag alone (3 pM vs. 3 nM). The porous and flexible ZIF-8 layer enabled efficient preconcentration of thiram in various real samples, achieving detection limits as low as 0.04 nM via soaking in lake water, 0.1 ng/cm^2^ on apple peel via swabbing, and rapid filtration-based detection in peach juice within 1 min. This post-synthetic adjustment of MOF properties not only enhanced molecular recognition and local analyte enrichment but also improved substrate stability, reproducibility, and size-selective response. These results underscore the importance of MOF functional tuning in achieving high-performance, real-world SERS sensors.

A notable example of post-synthetic modification in MOF-based sensors is the development of an aluminum-based MOF, HPDTP-Al [84]. This sensor was fabricated by functionalizing NH_2_-MIL53(Al) through diazotization, followed by coupling with resorcinol, introducing selective recognition sites for target metal ions. The modified MOF demonstrated high selectivity and sensitivity toward ultra-trace concentrations of Co^2+^ and Pd^2+^ ions, which were detected using spectrophotometric and fluorometric methods. Optimization of detection conditions enabled rapid response, with stable spectroscopic signals achieved in under one minute. The functionalized MOF also exhibited excellent recyclability, maintaining efficient performance over six cycles through regeneration with 0.1 M HCl and 0.1 M citrate solutions. Importantly, this PSM-enabled MOF demonstrated practical applicability in real-world scenarios, including the removal of Co^2+^ from electroplating wastewater and recovery of Pd^2+^ from electronic waste. This study exemplifies how surface functionalization and post-synthetic tuning can enhance MOF sensor performance, providing targeted molecular recognition, rapid detection, and reusability for environmentally and industrially relevant applications.

Post-synthetic modification of MOFs offers versatile opportunities to tailor their physical and chemical properties for enhanced device performance. As an example, Co/Zn bimetallic MOF nanosheets were coated onto flexible conductive fabrics to fabricate a highly flexible triboelectric nanogenerator (TENG), serving as both an energy-harvesting and sensing platform [85]. By systematically varying the Zn content from 0% to 50%, the electrical output was optimized, with 15% Zn providing the highest open-circuit voltage (47 V), short-circuit current (7 µA), and charge density (~17 nC/cm^2^), representing nearly a 450% improvement compared to the undoped material. The large surface area and tunable composition of the BMOF facilitated efficient mechanical-to-electrical energy conversion, while also enhancing gas-sensing performance, demonstrating selective response to ammonia at room temperature. This example illustrates how post-synthetic tuning of MOFs including compositional adjustment and surface engineering can extend their functionality beyond traditional adsorption or catalysis, enabling integration into flexible, multifunctional sensing devices.

In a representative example of post-synthetic tuning for enhanced bio-recognition, a magnetic MOF–SERS immunosensor was engineered through the integration of multiple functional layers, including Raman reporter loading, antibody conjugation, and plasmonic–magnetic hybridization [86]. Ag nanoparticles and the zeolitic imidazolate framework ZIF-67 were combined with rhodamine 6G (R6G) to form a SERS tag in which the MOF matrix improved analyte adsorption and contributed to the self-selective SERS enhancement, while R6G served as a stable Raman reporter. Concurrently, a CoFe_2_O_4_ magnetic core decorated with Ag nanoparticles was functionalized with human carboxylesterase 1 (hCE1) antibodies to create a magnetic capture substrate capable of rapid sample separation and uniform hotspot distribution. This hierarchical functionalization—encompassing reporter encapsulation, antibody grafting, and plasmonic–magnetic coupling—greatly amplified the SERS signal and improved reproducibility, enabling ultrasensitive quantification of hCE1 in complex biological samples with a detection limit of 3.6 pg/mL and a broad linear range spanning three orders of magnitude. This strategy exemplifies how surface functionalization and post-synthetic modification can be leveraged to create highly specific, separation-assisted, and reproducible MOF–SERS platforms for bioanalytical applications.

**Table 1:** Representative examples of MOF-based optical sensors for chemical and biological detection. Listed systems include their material type, target analyte, transduction mechanism, and analytical performance metrics.

Type of Material	Analyte	Detection Method	Enhancement Factor	RSD (%)	Sample Matrix	LOD (μM)	Linear Range (μM)	Ref.
ZIF-8@Ag heterostructure	4-Nitroaniline	Fiber-optic LSPR	5.91 × 10^8^	5.43	Aqueous model solution	1.13 × 10^−10^	10^−10^–10^−5^	[27]
Ag@UiO-66(NH_2_)/PDA-MIPs	Dye molecules	SERS	1.456 × 10^7^	2.94	River water	<0.0001	0.0001–0.01	[32]
Au@ZIF-8	Nitrofurantoin	SERS	110	9.45	Aqueous model solution	0.105	–	[45]
Au NFs@ZIF-67	Histamine	SERS	9.42 × 10^6^	4.62	Fish samples	87	10^3^–0.01	[50]
Zn-BTEC@ZIF-8	Histamine	Fluorescence	-	3.4	Water samples	10.305	14.1–2.820	[51]
Ag/ZIF-8	4-ATP	SERS	2.84 × 10^7^	6.99	Model analyte solution	0.001	–	[54]
Zn-MOF (HNU-55) + RhB	Tetracyclines	Ratiometric fluorescence	-	0.35	Lake water	0.0026–0.0075	–	[56]
Au NSs@LDH shell	R6G	SERS	-	8.9	Model solution	0.001	–	[60]
Au–Ag nanourchins + MOF@Au	Oxytetracycline	SERS	5.46 × 10^5^	4.32	Food samples	6.97 × 10^−9^	–	[61]
Cu-Ag@ZIF-8	PATP	SERS	-	12.7	Gas phase	0.000912	–	[64]
Ag@Au + 4-NPH @ ZIF-8	Acetone	SERS	-	-	Gas phase	0.101	–	[65]
MOF-199/Ag@Au	Dopamine	SERS	-	6.18	Human serum	0.000001	0.000001–1.0	[73]
AuNP/AE-MIL-101(Cr)	4-MPBA	SERS	-	-	Human serum	6.7 × 10^−7^	–	[74]
ZIF-8-derived ZnO + Ag NPs	R6G	SERS	1.8 × 10^8^	8.61	Model solution	10^−7^	–	[78]
MIL-101(Fe)@Ag	PATP, Formaldehyde	SERS	-	5.3	Aqueous solution	0.01; 0.001	–	[79]
AgMOFs on cotton swab	RDX, TNT	SERS	-	-	Solid surfaces	100.0	–	[80]
FP/Ag/ZIF-8	4-ATP, Thiram	SERS	-	7.98	Lake water	0.000003, 0.00004	–	[83]

## 3. Strategies for Engineering MOF-Based SERS Substrates

**Scheme 2:**
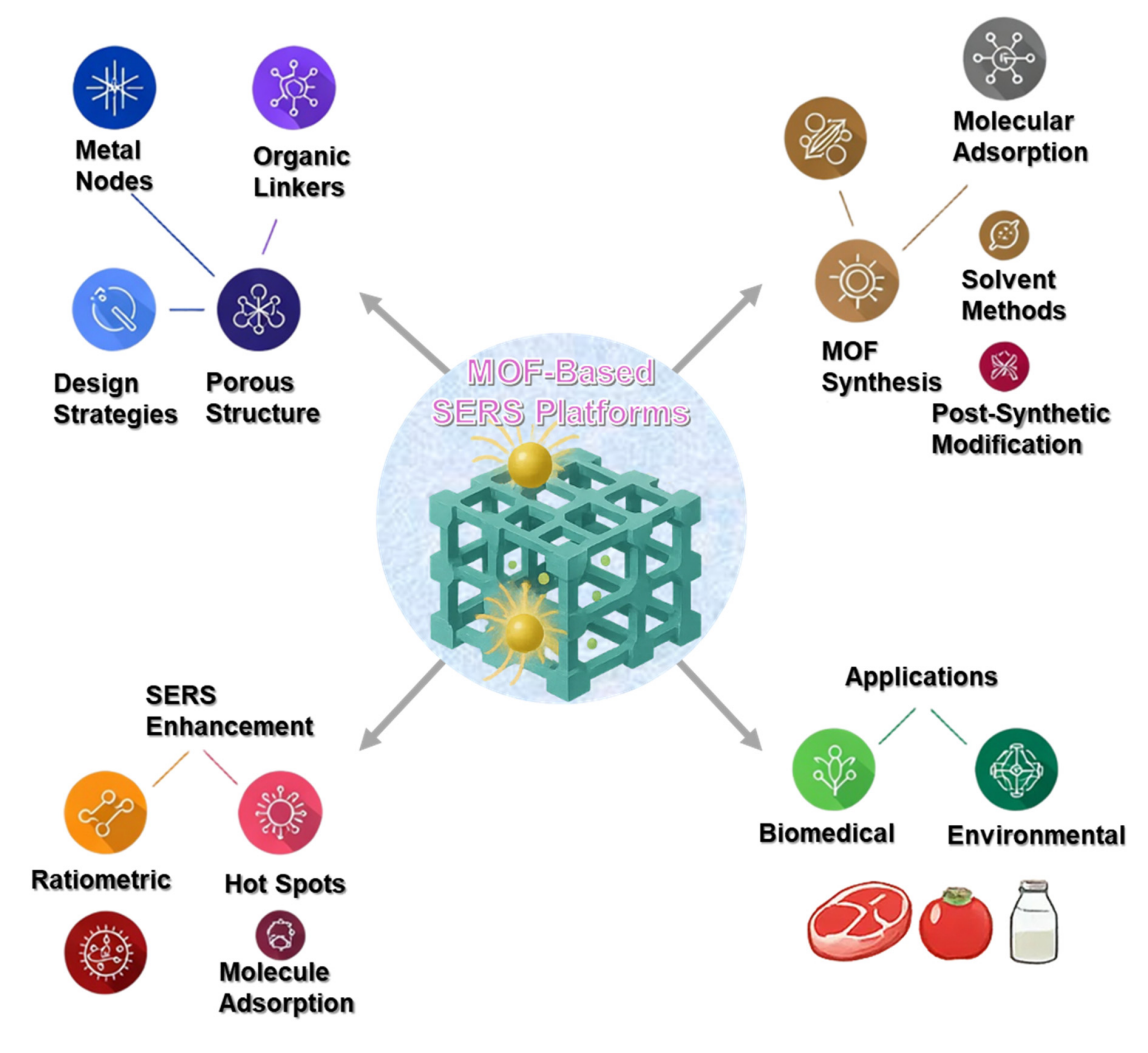
Conceptual map illustrating the major application domains of MOF-based SERS sensors (environmental, agricultural, food, and biomedical) and their corresponding MOF design strategies, functionalization approaches, and device formats.

The design of MOF-based SERS substrates requires careful optimization to balance key structural and functional parameters (Scheme 2). The materials need to deliver strong EM enhancement without losing what makes MOFs special their porosity, tunable chemistry, and structural order [87]. How well this balance is achieved largely determines the sensor’s sensitivity, selectivity, and reproducibility. Over the past few years, researchers have experimented with a wide range of strategies to fine-tune these systems tweaking interfaces, compositions, and architectures [88]. Most of these efforts fall into four general categories: metal–MOF composites, plasmon-free or charge-transfer active MOFs, chemical functionalization, and substrate fabrication techniques.

### 3.1. Metal–MOF Composites

Metal–MOF composites are probably the most familiar type of MOF-based SERS substrate. They combine the powerful plasmonic effects of noble metals with the selective adsorption and molecular recognition features of MOFs [89]. There are several ways to integrate metal nanoparticles (Au, Ag, Cu, etc.) into MOFs, and each has its own logic. In the in situ growth approach, metal precursors are introduced during or after the formation of the MOF. The particles then nucleate and grow inside the porous network, resulting in good contact between the two components and a fairly uniform dispersion of nanoparticles.

When these metal–MOF composite SERS platforms are compared side by side, clear design trade-offs emerge that extend beyond simple improvements in detection limits. AgNW@ZIF-67 substrates emphasize MOF thickness and pore accessibility as key parameters, where thinner coatings favor electromagnetic (EM) enhancement for large, surface-bound molecules, while thicker, porous shells promote charge-transfer (CT) contributions for smaller, diffusible analytes [90]. In contrast, MIL-101(Fe)-supported Au nanostar systems prioritize plasmonic geometry and interfacial charge transfer, achieving strong field localization through multibranched nanostructures but at the cost of more complex synthesis and reduced control over molecular sieving [91].

Zr-MOF–Au hybrids illustrate a different optimization strategy, where pore size matching and selective host–guest interactions are used to suppress matrix interference and enhance selectivity for bulky, hydrophobic pollutants such as dioxins, albeit with less emphasis on multifunctionality [92]. MOF-derived Ag–CuFe_2_O_4_ composites further highlight a trade-off between structural robustness and chemical enhancement, offering excellent recyclability and stability for environmental monitoring, but relying on high plasmonic loading rather than precise molecular recognition [93]. For uremic toxin sensing, AuNPs/ZIF-67/LIG platforms shift the design focus toward multimodal functionality, combining adsorption-driven SERS enhancement with electrochemical readout; however, this versatility comes at the expense of higher detection limits compared with SERS-optimized architectures [94].

Overall, these comparisons reveal that metal–MOF SERS sensor performance is governed by how plasmonic enhancement, pore accessibility, interfacial chemistry, and functional integration are balanced. Systems optimized for maximum sensitivity often sacrifice simplicity or selectivity, whereas multifunctional or matrix-tolerant designs prioritize robustness and application relevance over absolute signal amplification. Such trade-offs underscore the need for analyte- and application-specific design rather than universal optimization of detection limits alone.

### 3.2. Plasmon-Free and Charge-Transfer Active MOFs

Interestingly, not all SERS-active materials need to rely on noble metals. A growing number of MOFs have shown measurable Raman enhancement purely through charge-transfer mechanisms, even without any plasmonic component [95].

In these systems, light excitation promotes electronic coupling between the MOF and the adsorbed molecule, allowing charge transfer that modulates the Raman polarizability. MOFs with redox-active or π-conjugated ligands such as porphyrins, tetrathiafulvalene (TTF), or catecholates tend to be good candidates. Likewise, frameworks containing transition-metal centers like Cu, Fe, or Ti can help facilitate these processes. For example, conductive frameworks such as Cu_3_(HHTP)_2_ or Fe–TCPP-based MOFs have demonstrated modest but clear SERS enhancement, mainly because their extended conjugation and charge-transport channels make electron transfer easier. While their enhancement factors are generally lower than those of gold- or silver-based substrates, these plasmon-free systems have other advantages: they are less expensive, typically more biocompatible, and avoid background fluorescence. That combination makes them appealing for biosensing or even in vivo studies where metallic nanoparticles might be undesirable. That said, the field is still growing, and understanding the precise charge-transfer pathways and how to control them remains an open question.

MOFs, with their crystalline porous architectures and tunable metal–ligand networks, have emerged as promising substrates for plasmon-free, charge-transfer-driven SERS detection. Their high surface area, functionalizable pore interiors, and structural versatility allow enhanced interaction with target molecules, improving sensitivity and anti-interference performance compared with conventional noble metal-based SERS substrates. To further boost SERS activity, we developed an in situ ZIF-67/Co(OH)_2_ heterojunction on a nanocellulose paper platform, achieving a limit of detection (LOD) of 0.98 nmol/L for Rhodamine 6G and a Raman enhancement factor of 1.43 × 10^7^—approximately 100-fold higher than that of pure ZIF-67 [96]. This heterojunction approach was extended to other MOFs, including an in situ HKUST-1/Cu(OH)_2_ nanoplate, demonstrating that heterojunction formation facilitates efficient photoinduced charge transfer and significantly improves SERS performance. Application of this platform to pharmaceutical screening, exemplified by omeprazole detection, showed excellent sensitivity, tailorability, and anti-interference capability. These findings highlight that engineering MOF–heterojunction interfaces provides a versatile strategy for developing plasmon-free, charge-transfer-active SERS substrates with high reproducibility and practical applicability.

Recently, a CoOOH/ZnCdS-Vs heterostructured sensor was developed as a plasmon-free, charge-transfer-active platform through heterojunction engineering and defect modulation [97]. An S-type heterojunction formed between CoOOH and ZnCdS, coupled with sulfur vacancies introduced by annealing, effectively tuned the Zn–S and Cd–S bond strengths and promoted photogenerated electron–hole transfer. This synergistic design endowed the material with a high photocurrent density of 11.17 mA/cm^2^ at 1.23 V versus RHE. The sensor was subsequently applied for photoelectrochemical detection of Cu^2+^ and D-penicillamine (D-PA), where Cu^2+^ was reduced to Cu^+^ and Cu^0^, forming a CoOOH/ZnCdS/Cu_2_S heterojunction that decreased the photocurrent, while D-PA restored it through chelation via its mercapto and amino groups. The fabricated sensor demonstrated excellent selectivity, long-term stability with negligible photocurrent attenuation over 15 days at room temperature, and ultralow detection limits of 0.12 nM for Cu^2+^ and 1.26 µM for D-PA, highlighting the potential of charge-transfer–driven MOF-inspired systems for highly sensitive and stable sensing applications.

### 3.3. Functionalization and Surface Engineering

Fine-tuning a MOF’s chemistry is one of the simplest yet most powerful ways to improve its sensing performance. Functionalization can introduce specific sites that bind to certain molecules amine (–NH_2_), carboxyl (–COOH), or thiol (–SH) groups are popular choices [98]. These functional groups improve affinity toward pesticides, biomolecules, or heavy metal ions, depending on the target of interest. Post-synthetic modification (PSM) is especially useful because it allows researchers to alter the surface chemistry without compromising the MOF’s structure. Beyond chemical modification, doping and defect engineering offer another layer of control. By introducing heteroatoms or creating unsaturated metal sites, it is possible to tweak the electronic structure of the framework and boost charge-transfer efficiency [99]. This approach can yield noticeably stronger Raman signals. There is also growing interest in hybrid systems where MOFs are coupled with biorecognition elements aptamers, antibodies, or enzymes. These hybrids bring molecular specificity into play, which is particularly valuable when detecting pathogens or biomarkers in complex biological or environmental samples.

**Figure 3:**
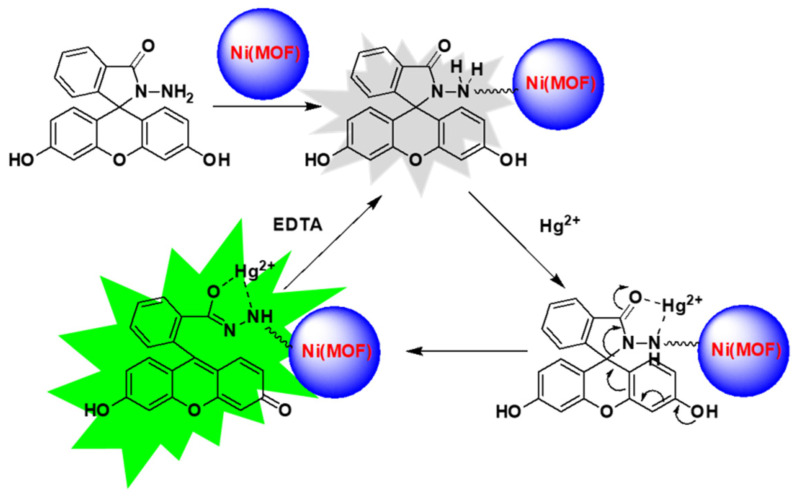
Schematic representation illustrating the sensing mechanism of Hg^2+^ detection by the fluorescein hydrazide-functionalized Ni(MOF) (FH@Ni(MOF)). Reproduced with permission from MDPI.

Recently, a fluorescein hydrazide-functionalized Ni(MOF), [Ni_3_(BTC)_2_(H_2_O)_3_]·(DMF)_3_(H_2_O)_3_, denoted as FH@Ni(MOF), was synthesized to achieve selective and sensitive detection of Hg^2+^ ions [100]. Functionalization with fluorescein hydrazide enabled the MOF to act as a highly selective optical sensor (Figure 3), producing a new absorption peak at 583 nm accompanied by a visible pink chromogenic change upon Hg^2+^ binding, while simultaneously enhancing fluorescence with a green emission. The binding constant of the FH@Ni(MOF) composite was determined to be 9.4 × 10^5^ M^−1^, with a detection limit of 0.02 μM (5 ppb). Importantly, the sensor exhibited good reversibility, sustaining performance over seven consecutive cycles, and successfully detected Hg^2+^ ions in practical water samples, including groundwater, tap water, and drinking water, demonstrating the effectiveness of surface functionalization and chemical engineering for improving selectivity, sensitivity, and practical applicability of MOF-based sensing platforms.

### 3.4. Substrate Fabrication and Integration Techniques

Even the most well-designed MOF–SERS material is only as useful as the substrate it is placed on. For real sensing applications, uniformity, reproducibility, and scalability are the major concerns. A number of fabrication methods have been adopted: drop-casting, spin-coating, layer-by-layer assembly, and electrochemical deposition are among the most common [101]. These techniques are simple enough to apply across different support materials glass, silicon wafers, paper, and flexible polymer films.

Recently, self-assembly and micro/nano-patterning approaches have attracted attention for their ability to produce large-area substrates with well-ordered hotspots and consistent SERS performance. Meanwhile, the integration of MOF–SERS composites into paper-based or textile platforms has opened up opportunities for low-cost, portable, and disposable sensors useful for on-site testing in agricultural fields or food safety inspections. At the more advanced end, some groups have incorporated these materials into microfluidic systems, enabling real-time analysis with controlled sample flow and minimal reagent consumption. These device-level integrations bring MOF–SERS technology closer to practical deployment rather than remaining just a proof of concept in the lab.

**Figure 4:**
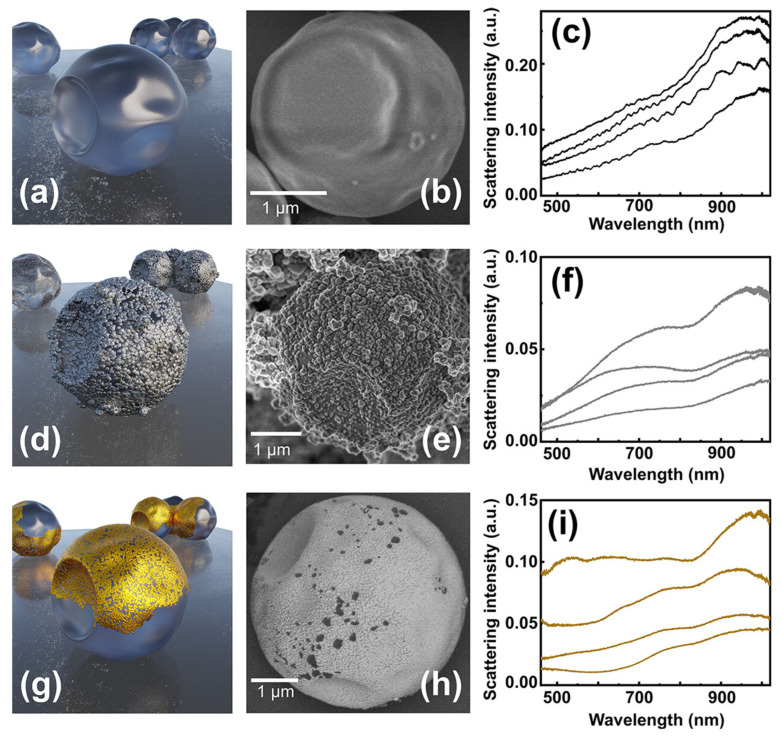
Schematic representations and SEM images of mercapto-functionalized silica microcarriers (SMPs) and their metal-decorated counterparts. (**a**) Schematic illustration (not to scale) of mercapto-functionalized bare silica microspheres (SMPs) and (**b**) corresponding SEM image, (**c**) Dark-field scattering spectra of bare SMPs showing a dominant resonance around ~950 nm with a weak shoulder near ~720 nm, attributed mainly to Mie scattering; (**d**) Schematic representation and (**e**) SEM image of SMPs decorated with Ag nanoparticles (NPs), (**f**) Representative scattering spectra of Ag-functionalized SMPs displaying multiple broad plasmonic resonances between ~600 and 750 nm, arising from size/shape variations and strong interparticle coupling, while retaining the SMP scattering feature near ~950 nm; (**g**) Schematic representation and (**h**) SEM image of SMPs sputtered with Au, (**i**) Scattering spectra of Au-functionalized SMPs showing a pronounced and reproducible plasmon resonance centered at ~760 nm, attributed to coupled Au nano-islands, along with weaker higher-energy features and the underlying SMP resonance at ~950 nm. Reproduced with permission ACS [102].

Recently, Mukherjee et al. reported a straightforward approach has been developed to fabricate three-dimensional (3D) SERS substrates by employing chemically modified silica particles as microcarriers onto which metal nanoparticles (NPs) are attached (Figure 4) [102]. Silver nanoparticles ranging from approximately 10 to 400 nm are generated via Tollens’ reagent on mercapto-functionalized silica particles, while gold nanoparticles with uniform island-like morphology are deposited through sputtering. Both types of substrates exhibit broad plasmon resonances within the visible spectral range, yielding significant SERS enhancement, with enhancement factors reaching up to 25. The 3D arrangement of NPs provides a substantially larger active surface area compared to traditional 2D substrates, enabling stronger SERS signals from analytes in flowing liquids or within cells and tissues, and demonstrating the advantages of integrating 3D architecture in substrate fabrication for enhanced analytical performance.

Moreover, an environmentally friendly approach was developed to fabricate a cost-effective plasmonic paper for SERS applications, achieved by the in situ growth of gold nanoparticles (AuNPs) on filter paper (FP) [103]. The substrate preparation utilized a double-layer biopolymer coating of chitosan (CS) and alginate (ALG) applied via layer-by-layer assembly through electrostatic interactions, which enhanced the reduction capability compared to single-layer coatings and facilitated dense AuNP formation. The resulting plasmonic paper exhibited excellent SERS performance, achieving an enhancement factor of 5.7 × 10^10^ and a low limit of detection of 1.37 × 10^−12^ M for 4-mercaptobenzoic acid (4-MBA). In addition, it demonstrated high spot-to-spot reproducibility with a relative standard deviation of 8.2% and long-term stability over 50 days. This substrate was successfully applied for detecting melamine in milk with a detection limit of 0.2 ppb and enabled simultaneous detection of β-agonists, including ractopamine and salbutamol, highlighting both its multiplexing capability and versatility. Overall, this fabrication strategy illustrates how simple, green, and integrated approaches can produce stable, high-performance SERS substrates suitable for practical analytical applications.

A novel three-dimensional (3D) SERS substrate was fabricated by integrating Au-shell Ag-core nanospheres (Au@Ag NSs) onto a pyramidal pitted silicon (PPSi) array with controllable size and arrangement, achieving a highly uniform and reproducible platform [104]. This composite substrate demonstrated excellent enhancement performance, as evidenced by the detection of Rhodamine 6G (R6G) down to 10^−9^ M, with an analytical enhancement factor (AEF) of 4.2 × 10^8^. The substrate also exhibited remarkable spectral uniformity, enabling quantitative SERS analysis of biological samples, including Staphylococcus aureus, with a correlation coefficient (R^2^) of 99.7%. Finite-difference time-domain (FDTD) simulations confirmed the distribution of strong local EM fields within the 3D structure, validating the hotspot formation and enhancement mechanism. These results indicate that the Au@Ag NSs/PPSi substrate, with its tunable architecture and integrated plasmonic nanostructures, is a promising platform for sensitive, label-free detection and represents a practical approach for fabricating robust, high-performance SERS-active devices.

A novel silicon (Si) substrate was fabricated using vortex femtosecond laser beams in ambient air, presenting an innovative platform for highly sensitive and reusable SERS applications [105]. The laser processing produced composite nanostructures with bush-like formations atop elongated features, resulting from the orbital angular momentum of the vortex beam. Gold nanoparticles were subsequently deposited onto the substrate, creating densely distributed hotspots, as confirmed by COMSOL multiphysics 5.5 simulations, which revealed enhanced local EM fields compared to substrates processed with conventional Gaussian beams. This engineered substrate demonstrated exceptional SERS performance, achieving limits of detection of 3.91 pM for malachite green and 2.69 pg·mL^−1^ for microcystin-LR, while exhibiting remarkable stability, reusability, and temporal resistance. These results highlight the effectiveness of advanced laser-fabricated nanostructures in producing robust, high-performance SERS substrates, emphasizing the critical role of precise fabrication and integration techniques in enhancing analyte detection.

A flexible and transparent tape-based SERS substrate was fabricated by sandwiching electrodeposited silver nanoparticles (AgNPs) between a polyimide (PI) tape and a graphene (G) layer [106]. The graphene layer was prepared via chemical vapor deposition (CVD) on a copper substrate, followed by the electrodeposition of AgNPs and subsequent transfer onto the PI tape to form the G/AgNPs/PI heterostructure. Methyl parathion (MP) was employed as a probe molecule to evaluate SERS performance, achieving a detection limit of 68 ng/cm^2^. The inclusion of the graphene layer improved the stability and protection of the AgNPs, extending the substrate’s shelf life at room temperature to over 48 days, compared to 27 days for a substrate without graphene. The practical utility of this tape-based substrate was demonstrated by detecting MP residues on apple surfaces, with both laser excitation and Raman signal collection performed through the tape side, highlighting its potential for flexible, integrable, and real-world SERS applications.

Achieving reproducible and high-performance SERS substrates remains a critical challenge due to nanoscale variations in plasmonic hotspot geometries. To address this, Yun et al. reported a silver-based nanolaminate substrate featuring vertically stacked 3D nanogaps, which enables the formation of densely packed and uniform hotspots [107]. By selectively etching dielectric layers, the nanogap regions are exposed to analyte molecules, resulting in a substantial enhancement factor of 1.75 × 10^8^. The substrate demonstrates excellent uniformity, with a relative standard deviation of 11% across 400 measurement points, confirming reproducible SERS performance. Furthermore, employing high-throughput nanoimprint lithography allows the fabrication of large-area devices (~16 cm^2^) in a cost-effective manner, making this nanolaminate SERS substrate highly suitable for practical sensing applications requiring both sensitivity and reproducibility.

SERS offers high sensitivity, nondestructive analysis, and molecular fingerprinting, making it highly promising for point-of-care testing (POCT). However, its practical application is often limited by challenges in rapidly producing substrates with high reproducibility, uniformity, and signal enhancement. To address this, a one-step chemical printing approach was developed to fabricate three-dimensional (3D) plasmon-coupled silver nanocoral (AgNC) substrates within approximately 5 min, without requiring pretreatment or complex instrumentation [108]. The galvanic replacement reaction between AgNO_3_ and Cu sheets generates Ag^0^ for nanostructure formation and Cu^2+^ to facilitate polymerization of fish sperm DNA (FSDNA), which crosslinks to protect AgNCs and control their coral-like morphology. The resulting substrates exhibit strong 3D plasmonic coupling both between nanocoral tentacles and between nanocorals and Cu sheets, producing an enhancement factor of 1.96 × 10^8^ and excellent uniformity with a relative standard deviation below 6%. These AgNC substrates were successfully applied to quantify weak-affinity food colorants, including Brilliant Blue, Allura Red, and Sunset Yellow, with detection limits of 0.053, 0.087, and 0.089 ppm, respectively, and recoveries in complex food and urine samples ranging from 91 to 119 facile and rapid fabrication strategy demonstrates the potential for producing robust, high-performance SERS substrates suitable for on-site food safety monitoring and POCT applications.

## 4. Applications of MOF-Based SERS Sensors

The practical value of MOF-based SERS sensors really becomes clear when looking at their diverse applications across environmental, agricultural, and biomedical fields [109]. By integrating porous molecular enrichment, tailored surface chemistry, and plasmonic signal amplification, these hybrid platforms address many limitations of conventional SERS substrates when applied to complex real-world matrices [110]. While many systems remain at the proof-of-concept stage, recent studies increasingly focus on robustness, reproducibility, and deployability, indicating a gradual transition toward field-relevant sensing technologies.

### 4.1. Environmental Monitoring

#### 4.1.1. Heavy-Metal and Inorganic Pollutant Detection

Environmental monitoring of toxic metal ions requires sensing platforms capable of selective enrichment and interference tolerance in chemically complex water matrices. MOF-integrated SERS substrates are particularly effective in this context due to their high surface area and affinity-driven adsorption mechanisms [111]. There is a growing recognition that the next stage of development will require not just material innovation but also better surface cleaning strategies and integrated sample pretreatment steps.

A representative example is the Ag@Fe_3_O_4_/UiO-66-NH_2_ (Ag@FUN) magnetic composite, which combines UiO-66-NH_2_ for selective Cr(VI) adsorption with Ag nanoparticles for SERS enhancement [112]. Control over Ag loading enabled tunable sensitivity while preserving signal reproducibility and storage stability. The resulting composites exhibited strong and reproducible SERS signals, with detection limits as low as 7.81 × 10^−8^ M in tap water and 8.89 × 10^−8^ M in pond water, along with excellent storage stability. Beyond detection, the same material facilitated photocatalytic Cr(VI) reduction under simulated sunlight, demonstrating multifunctionality through coupled sensing and remediation. The high performance is attributed to the porous MOF structure, which promotes Cr(VI) adsorption through Zr–O coordination, electrostatic interactions, and pore-filling effects, concentrating analytes near the plasmonic hotspots.

#### 4.1.2. Pesticide and Organic Contaminant Detection

For organic contaminants such as pesticides, MOFs primarily serve as molecular sieves and protective scaffolds that stabilize plasmonic components while improving analyte accessibility. An Ag@ZIF-8@Au hybrid platform exemplifies this strategy, where ZIF-8 prevents Ag oxidation and enhances analyte enrichment, while Ag–Au plasmonic coupling generates dense electromagnetic hotspots [113]. This architecture enabled reproducible detection of acetamiprid across diverse environmental and agricultural matrices. The platform demonstrated high sensitivity with a limit of detection of 9.03 × 10^−10^ M and a SERS enhancement factor of 4.3 × 10^7^, alongside excellent reproducibility (6.50% and 7.20% RSD for 30 randomly selected points) and long-term stability (3.13% RSD over six weeks).

Across environmental applications, two dominant design strategies emerge: (i) adsorptive MOFs paired with magnetic or photocatalytic functionality (e.g., Ag@FUN) to address both detection and remediation, and (ii) plasmon-protected MOF architectures (e.g., ZIF-8-based hybrids) that prioritise long-term signal stability and matrix compatibility. While multifunctional systems offer broader utility, they often require more complex fabrication, whereas simpler MOF–plasmonic hybrids favour scalability and routine deployment. These trade-offs highlight that application context, rather than absolute sensitivity, should guide material selection.

### 4.2. Agricultural and Food Analysis

#### 4.2.1. Food Freshness and Spoilage Monitoring

Real-time assessment of food freshness demands non-destructive sensing approaches capable of detecting volatile compounds and pH changes. The AuNS@ZIF-8-SLIPS platform addresses this challenge by integrating ZIF-8-encapsulated gold nanostars into a slippery liquid-infused porous surface, enabling efficient capture of gaseous spoilage markers [114]. To address this, a slippery liquid-infused porous surface (SLIPS) platform was developed to enable real-time SERS monitoring of gaseous molecules associated with shrimp spoilage. In this approach, 4-mercaptopyridine (4-Mpy) and 4-mercaptobenzaldehyde (4-MBA)-functionalized ZIF-8-encapsulated gold nanostars (AuNS@ZIF-8) were employed as responsive probes to detect pH changes and volatile biogenic amines, respectively. The inherent porosity of ZIF-8 facilitated efficient trapping and preconcentration of gaseous analytes, while the SLIPS substrate enhanced enrichment at the plasmonic hotspots. This combination resulted in a highly sensitive and reproducible sensing platform, with detection ranges of 4.0–9.0 for pH and 10^−7^–10^−3^ (*v*/*v*) for biogenic amines, and relative standard deviations of 4.1% and 4.2%, respectively. The system was successfully applied to monitor shrimp spoilage under different storage conditions (25 °C and 4 °C) in real time, demonstrating its potential as a non-destructive, rapid, and accurate tool for food freshness evaluation.

#### 4.2.2. Agrochemical, Mycotoxin, and Multi-Residue Detection

Monitoring chemical residues in food and agricultural products requires sensing platforms that combine high molecular selectivity, strong enrichment capability, and tolerance to complex sample matrices. MOF-based SERS sensors are particularly well suited for these tasks due to their tunable pore structures, surface functionality, and compatibility with plasmonic nanomaterials [115].

A nanoscale-engineered ZIF-67@AgNPs@PDA triple-layer composite illustrates how MOF scaffolds can be combined with plasmonic nanoparticles and protective polymer coatings to enable reliable detection of both pesticides and veterinary drugs [116]. This hybrid platform functions as a highly efficient SERS-active substrate, allowing detection of THR and CAP at characteristic Raman bands of 1378 and 1596 cm^−1^, respectively. The enhanced sensitivity toward THR is attributed to S–S bond cleavage and monolayer adsorption, whereas CAP exhibited weaker interactions and multilayer adsorption. The ZIF-67@AgNPs@PDA substrate achieved ultratrace detection limits of 3.2 × 10^−13^ M for THR and 7.7 × 10^−12^ M for CAP, surpassing many previously reported nanomaterial-based sensors. Importantly, this SERS system maintained reliable performance in real samples including tap water, apple juice, and pork demonstrating its robustness and potential for practical applications in food safety and agricultural monitoring.

Beyond single-analyte sensing, MOF-imprinted and flexible sensing platforms have been developed to support rapid, on-site analysis. A paper-based MOF sensor with molecularly imprinted recognition sites enabled selective thiacloprid detection while minimizing signal variability and coffee-ring effects, further enhanced by smartphone-assisted image processing for field deployment [117]. Such designs prioritise portability and user accessibility over maximum sensitivity, addressing practical constraints in agricultural monitoring.

For highly regulated contaminants such as mycotoxins, magnetic MOF-based ratiometric SERS aptasensors provide improved accuracy and interference resistance. A magnetic MOF system integrating AuMBA@Ag substrates and aptamer-modified capture probes enabled sensitive patulin detection in apple samples through ratiometric signal analysis, effectively compensating for matrix-induced fluctuations [118]. The system integrates magnetic MOFs loaded with 4-mercaptobenzoic acid-labeled AuMBA@Ag as the SERS-active substrate and gold nanorods modified with Rhodamine 6G and aptamers as capture probes. This design enhances sensitivity by combining magnetic separation with abundant SERS hotspots from the MOF nanocomposites. The ratiometric SERS signal exhibited a negative correlation with PAT concentrations over a range of 0.01–100 ng/mL, achieving a limit of detection of 0.0465 ng/mL. Recovery studies in apple samples demonstrated excellent accuracy and anti-interference capability, with recoveries between 95.90% and 105.83%. These results highlight the strong potential of this magnetic MOF-based SERS aptasensor for reliable mycotoxin monitoring in real food matrices.

Extending this strategy to clinical relevance, a Au CRDs@ZIF-67/MCE microdevice enabled simultaneous detection of multiple pesticide residues in human serum and urine without sample pretreatment, demonstrating the feasibility of rapid point-of-care diagnostics for mixed-exposure scenarios [119]. The device employs a multifunctional Au CRDs@ZIF-67/MCE composite, where a porous cellulose ester membrane is modified with ZIF-67 and concave rhombic dodecahedral gold nanoparticles, serving both as a SERS-active substrate and analyte sorbent. Assembled in a 24-well plate, the system enhances enrichment selectivity via a “1 + 1 dual-mode separation” effect, while the concave tips of the Au CRDs amplify SERS signals. Quantitative analysis showed strong correlations between SERS intensity and analyte concentration (R-values: 0.911–0.986) with detection limits ranging from 0.16 to 2.71 ppb. Importantly, patient bio-samples were analyzed in just 5 min without any sample preparation, demonstrating the device’s high sensitivity, reproducibility, and potential as a user-friendly, rapid POCD tool for multi-pesticide poisoning scenarios.

Across agrochemical, mycotoxin, and multi-residue applications, MOF-based SERS sensors reveal distinct design trade-offs between sensitivity, multiplexing capability, and operational simplicity. Highly engineered nanocomposites offer superior sensitivity and matrix tolerance but require multistep fabrication. In contrast, paper-based and membrane-integrated platforms favour scalability, portability, and rapid analysis at the expense of ultimate sensitivity. A comparative overview of representative MOF architectures, sensing modes, and analytical performance is summarized in Table 2. These comparisons underscore that application-driven considerations such as regulatory thresholds, sample throughput, and field usability should guide MOF–SERS sensor design, rather than detection limits alone.

## 5. Applications in Biomedicine

MOF-based SERS sensors have emerged as powerful analytical platforms in biomedicine due to their ability to integrate high electromagnetic enhancement, selective molecular enrichment, and tunable surface chemistry within a single architecture [120]. Compared with conventional diagnostic approaches that often require extensive sample preparation and centralized instrumentation, MOF–SERS systems enable rapid, label-free detection with reduced interference from complex biological matrices such as blood, serum, or food extracts [121]. The intrinsic porosity of MOFs, combined with their compatibility with plasmonic nanostructures and magnetic components, has enabled diverse biomedical applications ranging from pathogen identification and toxin monitoring to therapeutic drug analysis and theranostics.

### 5.1. Pathogen Detection

MOF-based SERS sensors have been extensively explored for pathogen detection owing to their ability to selectively enrich microbial biomarkers and localize them near plasmonic hotspots [122]. MOF-based SERS platforms offer distinct advantages in this context by enabling target preconcentration near plasmonic hotspots while simultaneously supporting selective recognition through aptamers, antibodies, or lectins.

#### 5.1.1. Bacterial Pathogen Sensors

Several MOF-integrated SERS substrates have been developed for sensitive bacterial detection by combining electromagnetic amplification with molecular enrichment. A PDMS-MXene@MOF@Ag ternary substrate demonstrated effective detection of *Escherichia coli* by leveraging synergistic chemical enhancement from MXene, adsorption capability from the MOF, and strong plasmonic activity from Ag nanoparticles [123]. This multi-component architecture enabled bacterial detection across a wide dynamic range (9 × 10^2^–9 × 10^10^ CFU mL^−1^) with excellent reproducibility and stability.

Multiplexed bacterial detection has also been achieved through immuno-SERS strategies. Yang et al. developed a sandwich-type immunosensor using lectin-functionalized magnetic nanoparticles for bacterial capture and COF-based Raman nanotags for signal amplification, enabling simultaneous detection of multiple foodborne pathogens with limits of detection down to 10^1^ CFU mL^−1^ using a portable Raman system [124]. In contrast, Cai et al. employed an AuAg-doped Prussian Blue MOF exhibiting ultrahigh peroxidase-like activity, enabling dual-mode colorimetric and SERS detection of *E. coli* and *S. aureus* with detection limits as low as 6 CFU mL^−1^ [125].

Across these bacterial sensing platforms, MOFs serve distinct but complementary roles: adsorption scaffolds (PDMS-MXene@MOF@Ag), signal-amplifying nanozymes (AuAg@PB MOF), or carriers for Raman reporters (COF nanotags). Magnetic separation enhances robustness in complex matrices, while dual-mode readouts improve reliability. However, increased architectural complexity often trades off with fabrication simplicity, underscoring the need to balance sensitivity, scalability, and operational practicality.

#### 5.1.2. Bacterial Toxins and Mycotoxins

MOF-based SERS systems have also been extensively applied to toxin detection, where high sensitivity and matrix tolerance are critical. A NiRs@MOF-74(Ni)/Ag magnetic substrate enabled simultaneous detection of T-2 toxin and deoxynivalenol with sub-μg L^−1^ limits of detection, showing strong agreement with HPLC-MS analysis [126]. Label-free detection of staphylococcal enterotoxin C (SEC) was achieved using solid Au–Ag Janus@Au nanoparticles, where plasmonic coupling and Ag protection improved both sensitivity and stability, yielding a detection limit of 0.55 pg mL^−1^ [127].

Dual-mode platforms further expand analytical reliability. Mn/Fe-MIL(53)@Au nanostar-based aptasensors enabled combined colorimetric and SERS detection of Shiga toxin II, improving dynamic range and analytical confidence in milk samples [128]. Similarly, multifunctional magnetic composites integrating MOFs, gold nanostructures, and aptamers enabled sensitive detection and simultaneous photothermal sterilization of *Vibrio parahaemolyticus*, demonstrating detection limits below 10 CFU mL^−1^ alongside rapid bacterial inactivation [129].

Toxin-oriented MOF–SERS systems highlight the importance of adsorption efficiency and signal amplification over whole-cell capture. Magnetic MOF composites improve matrix compatibility, while dual-mode sensing mitigates false positives. Notably, platforms integrating therapeutic functions (e.g., photothermal sterilization) illustrate how MOF–SERS architectures can transition from passive sensing to active intervention.

#### 5.1.3. Mechanistic Insight and Operando Biosensing

Beyond analytical performance, understanding interfacial biosensing mechanisms is critical for reliable pathogen detection. An operando PEC-SERS platform employing porphyrin-based Zr-MOFs enabled simultaneous acquisition of photocurrent and SERS signals, revealing real-time conformational changes during aptamer–target binding [130]. This dual-signal strategy exposed discrepancies inherent to conventional electrochemical measurements and improved analytical accuracy for patulin detection.

Operando MOF-based SERS systems uniquely provide mechanistic transparency, allowing correlation between electron transfer and molecular recognition. While technically complex, such approaches offer a pathway toward self-validating biosensors with improved reliability in real-world biomedical applications.

### 5.2. Biomarker and Drug Monitoring

MOF–SERS platforms developed for biomarker monitoring further illustrate how enrichment strategies influence analytical performance [131]. In contrast to proof-of-concept sensing, clinically relevant applications require reproducible quantification and resistance to biological interference.

**Figure 5:**
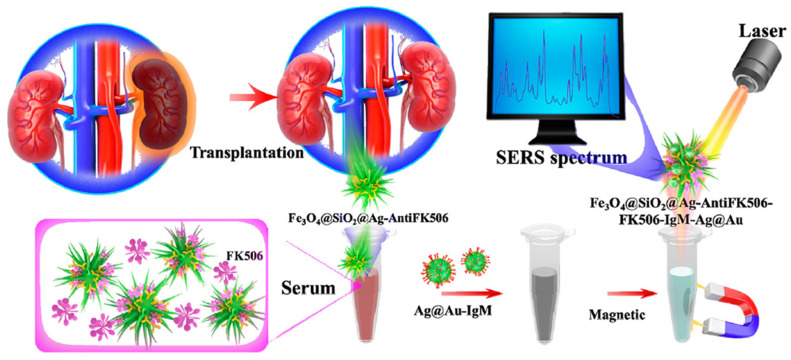
Schematic illustration of a magnetic plasmonic sandwich-type SERS biosensor for ultrasensitive detection of tacrolimus (FK506) in blood. The spiky Fe_3_O_4_@SiO_2_@Ag magnetic superstructure and hollow Ag@Au superstructure generate abundant plasmonic hotspots for enhanced SERS signals, while the magnetic feature enables target enrichment and separation. Reproduced with permission ACS [132].

A representative example is a magnetic plasmonic sandwich-type SERS biosensor developed for ultrasensitive detection of the immunosuppressive drug tacrolimus (FK506) [132]. The system integrates a spiky Fe_3_O_4_@SiO_2_@Ag magnetic superstructure with a hollow Ag@Au superstructure, generating abundant plasmonic hotspots while enabling magnetic enrichment and separation (Figure 5). This design achieved a low detection limit of 0.33 ng mL^−1^ and accurate quantification in patient blood samples, illustrating how multifunctional integration improves analytical reliability in real clinical matrices.

Aptamer-assisted histamine detection using IRMOF-3@Au/PDMS combined selective molecular recognition with flexible substrate design, enabling sensitive quantification in food matrices while maintaining operational simplicity [133]. In contrast, hierarchical porous hybrids integrating homochiral MOFs with mesoporous Au films achieved ultralow detection limits for chiral drugs through combined physical and chemical enhancement, albeit with increased fabrication complexity [134].

**Figure 6:**
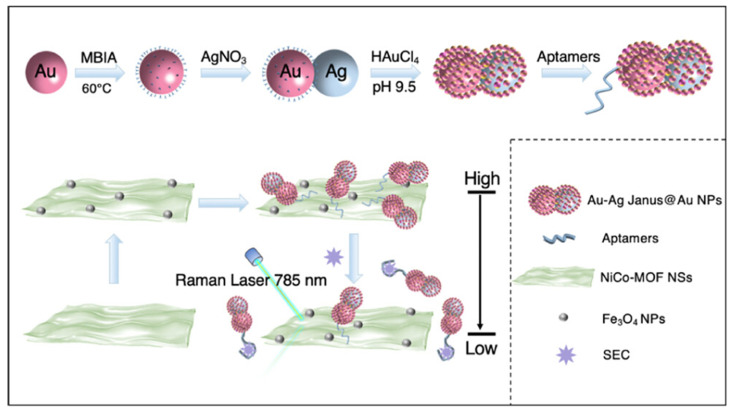
Schematic illustration of a label-free SERS aptasensor for sensitive detection of staphylococcal enterotoxin C (SEC) using solid Au–Ag Janus@gold nanoparticles (Au–Ag Janus@Au NPs). Reproduced with permission ACS [127].

Beyond drug molecules, MOF–SERS platforms have also been applied to toxin and biomarker detection using plasmonically engineered nanostructures. For instance, a label-free SERS aptasensor based on solid Au–Ag Janus@gold nanoparticles was developed for sensitive detection of staphylococcal enterotoxin C (SEC) in food matrices (Figure 6) [127].

Similarly, Mn/Fe-MIL(53)@Au nanostar systems combined colorimetric and SERS signals for Shiga toxin II detection, expanding the dynamic range and improving accuracy in milk samples [128]. In a related application-oriented approach, a multifunctional magnetic MOF-based composite, Fe_3_O_4_@MOF(Fe–Cu)–GNS–MBA–Apt, was developed for the simultaneous detection and in situ inactivation of *Vibrio parahaemolyticus* [129]. Here, aptamer-modified MOFs enabled selective pathogen capture from complex food matrices via magnetic separation, while gold nanostar–MOF hybrids provided both strong SERS signals and catalytic activity for colorimetric readout. Beyond detection, the composite materials also supported photothermal sterilization under near-infrared irradiation, enabling rapid elimination of the captured bacteria. Successful testing in fresh shrimp samples highlights the potential of such multifunctional MOF–plasmonic materials for integrated pathogen surveillance, food safety assurance, and environmental diagnostics.

A material-integrated SERS sandwich platform was reported for the practical detection of Salmonella Typhimurium in food samples, demonstrating how multifunctional nanomaterials can be engineered for real-world pathogen monitoring [135]. In this design, dendritic mesoporous silica carriers loaded with plasmonic nanoparticles and Raman reporters served as efficient signal amplification units, while covalent organic frameworks encapsulating magnetic Fe_3_O_4_ nanoparticles functioned as enrichment and separation components. The coordinated use of these materials enabled effective bacterial capture and hotspot formation, allowing reliable identification of *S. Typhimurium* at low concentrations. Importantly, the platform was validated in spiked chicken samples, underscoring its applicability for routine food safety inspection of raw meat products.

Magnetic MOF–plasmonic hybrids, such as NiRs@MOF-74(Ni)/Ag substrates, emphasize enrichment efficiency and matrix tolerance for toxin detection [126]. This system enabled simultaneous quantification of T-2 toxin and deoxynivalenol with results comparable to HPLC–MS.

Across pathogen-detection studies, trade-offs emerge between sensitivity, system complexity, and application robustness. Single-mode SERS substrates prioritize simplicity and speed, whereas dual-mode and operando systems improve reliability at the cost of increased fabrication complexity. Magnetic MOF–plasmonic platforms consistently enhance matrix tolerance and reproducibility, making them particularly suitable for real-world food safety and clinical diagnostics.

Magnetic MOF–plasmonic systems, such as Co-MOF-74@Au-based immunosensors, emphasize rapid separation, signal stability, and matrix tolerance for protein and drug monitoring [136]. These designs prioritize reproducibility and clinical robustness over absolute sensitivity, reflecting application-driven trade-offs.

SERS-active microneedles integrating Au@Ag nanostructures enable minimally invasive, real-time drug monitoring in dermal interstitial fluid (ISF), addressing key limitations of blood-based analysis [137]. Comparative pharmacokinetic results reveal strong drug-dependent ISF–blood correlations, highlighting ISF as a viable but selective surrogate for blood and underscoring the importance of tailoring in vivo SERS platforms to drug-specific transport behavior.

Taken together, biomarker and toxin sensing studies demonstrate that no single MOF–SERS design universally outperforms others. Instead, optimal performance arises from carefully balancing enrichment efficiency, plasmonic architecture, selectivity mechanisms, and system complexity according to the intended sensing scenario.

### 5.3. Imaging and Theranostics

Beyond sensing, MOF–SERS hybrids enable integrated imaging and therapeutic functions [138]. For example, Au@MOF hybrid systems incorporating photothermal agents or therapeutic drugs have been exploited for SERS-assisted cancer imaging and targeted treatment. The intrinsic porosity of MOFs enables regulated release of therapeutic cargos, whereas the SERS response offers real-time information on probe distribution and local concentration. Moreover, MOF-based SERS nanotags have been applied to high-contrast, multiplexed cellular imaging in vitro, enabling simultaneous and non-destructive visualization of multiple biomolecular targets [139]. Together, these multifunctional platforms highlight the potential of MOF–SERS hybrids for integrated diagnostic–therapeutic applications, although challenges related to biocompatibility, long-term stability, and in vivo translation remain to be addressed.

**Figure 7:**
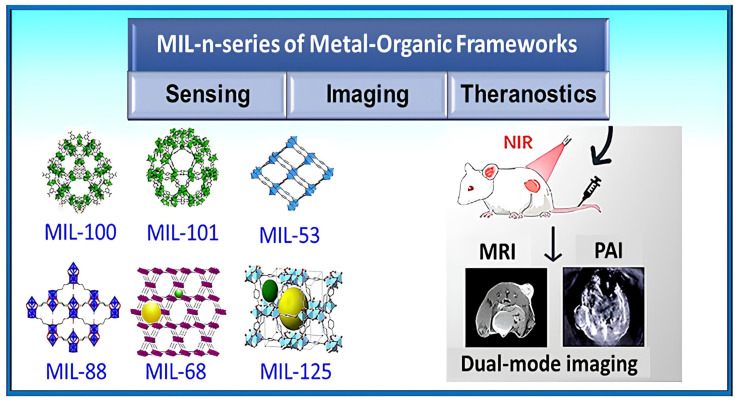
Schematic illustration of MIL-n MOFs as multifunctional platforms for cancer theranostics. Reproduced with permission ACS [140].

Au@MOF composites loaded with photothermal agents or drugs provide real-time SERS-guided imaging alongside controlled drug release. MIL-n MOFs, in particular, offer high porosity, biocompatibility, and structural tunability, supporting cancer diagnosis and theranostic applications (Figure 7) [140].

Plasmon-enhanced catalytic systems, such as Au nanorod/Fe-MOF hybrids, demonstrated photo-enhanced peroxidase-like activity alongside ultralow SERS detection limits, extending functionality toward cancer therapy and chemical sensing [141]. Targeted nanocarriers integrating Ag nanoparticles into folic-acid-functionalized MOFs further enabled selective cancer cell detection and therapy with minimal toxicity to normal cells [142].

As summarized in Table 3, theranostic MOF–SERS systems reveal a fundamental trade-off between multifunctionality and translational potential. While integrated imaging–therapeutic platforms enhance diagnostic capability, their sensing effectiveness strongly depends on the specific MOF architecture and integration strategy employed, with different MOF-based designs exhibiting varying performance toward distinct analyte classes. Persistent challenges related to biocompatibility, long-term stability, and limited in vivo validation continue to constrain clinical implementation.

## 6. Challenges and Future Perspectives

Although MOF-based SERS sensors have achieved significant progress in agricultural, biomedical, and environmental monitoring, several key obstacles still hinder their transition from laboratory research to real-world use. A major challenge is the difficulty in ensuring reproducible and scalable fabrication [143]. SERS performance depends critically on the size, density, and spatial distribution of plasmonic hotspots; even minor variations in nanoparticle loading, MOF crystallinity, or substrate deposition can lead to large signal fluctuations. While approaches such as standardized synthesis protocols, controlled in situ nanoparticle growth, automated fabrication, and emerging scalable techniques like roll-to-roll production show potential, consistent large-scale manufacturing suitable for clinical or industrial use remains challenging [144,145]. However, consistent large-scale production suitable for clinical or industrial application remains elusive.

Another key obstacle is the complexity of real-world sample matrices [146]. Biological fluids, food extracts, and environmental samples often contain multiple interfering compounds that compete for adsorption or produce overlapping Raman signals, complicating accurate trace-level detection [147]. Although MOFs provide selective adsorption and sieving capabilities, these alone are insufficient to eliminate all interference [148]. Strategies such as surface functionalization with recognition elements, sample pretreatment, and enrichment can mitigate matrix effects, but careful calibration and controlled handling remain essential for reliable practical measurements.

Quantitative SERS analysis presents additional difficulties due to variability in hotspot intensity, laser alignment, and analyte adsorption [149,150]. Robust quantification requires effective normalization strategies, such as embedding internal Raman reference molecules or integrating stable reporters into the MOF structure [151]. Statistical models and machine-learning tools are increasingly employed to compensate for spectral variability, though the inherent surface sensitivity of SERS means some uncertainty will always remain. The aim is therefore consistent and reliable trends rather than absolute precision.

Successful deployment also depends on integration with portable Raman devices for on-site or point-of-care use. This requires substrates that maintain performance under diverse environmental and operational conditions. Artificial intelligence can support this transition by automating spectral interpretation, reducing noise, detecting anomalies, and recognizing complex patterns, ultimately improving user-friendliness and reducing operator error [152]. Realizing fully integrated MOF–SERS platforms that combine stable substrates, portable spectrometers, and AI-assisted analytics will require extensive calibration, optimization, and field validation [153].

Finally, environmental and biocompatibility considerations are becoming increasingly important. The use of MOFs and metallic nanoparticles in agricultural and clinical environments raises concerns regarding toxicity, degradation byproducts, and long-term persistence. Developing biocompatible or biodegradable MOFs, adopting green synthesis protocols, and conducting comprehensive studies on environmental fate and biological impact will be essential to ensure safe and responsible implementation.

## 7. Conclusions and Future Outlook

MOF-based SERS sensors have clearly established themselves as a versatile platform for sensitive and selective detection across agricultural and biomedical fields. By marrying the ultrahigh sensitivity of plasmonic nanostructures with the tunable porosity and chemical functionality of MOFs, these hybrid systems allow effective analyte preconcentration, controlled hotspot formation, and molecular recognition—addressing many of the limitations seen in conventional SERS substrates. Advances in substrate engineering, including compositional tuning, ligand functionalization, defect or doping strategies, and hierarchical or core–shell architectures, have noticeably improved signal enhancement, selectivity, and operational stability.

In agriculture, MOF–SERS platforms have demonstrated practical utility for detecting pesticides, herbicides, heavy metals, mycotoxins, and other food contaminants, often achieving rapid, label-free detection in real samples. Similarly, in biomedicine, these systems show promise for pathogen detection, biomarker monitoring, therapeutic drug quantification, and even theranostic applications such as imaging-guided therapy. The integration of MOF–SERS sensors with portable Raman spectrometers, microfluidics, and AI-assisted spectral analysis has further broadened their potential, moving them closer to field-deployable or point-of-care use.

That said, challenges remain. Consistently reproducing these substrates at scale, mitigating matrix interference in complex samples, ensuring quantitative reliability, and addressing environmental and biocompatibility concerns are still significant hurdles. Translating lab-scale MOF–SERS platforms into robust, automated, multifunctional devices will likely require ongoing innovation in materials design, interface engineering, and system integration.

Looking ahead, several directions seem particularly promising:

- Rational design of multifunctional MOFs with controlled porosity, tunable functional groups, and optimized plasmonic interfaces to enhance sensitivity, selectivity, and analyte enrichment.
- Scalable fabrication techniques capable of producing uniform, reproducible, and cost-effective substrates for routine agricultural or clinical applications.
- Integration with AI and machine learning to support real-time spectral interpretation, automated quantification, and multiplexed detection in complex biological or environmental matrices.
- Development of biocompatible and environmentally benign MOFs, ensuring safe use in both biomedical and agricultural settings.
- Combination with advanced technologies, such as microfluidics, wearable devices, and theranostic platforms, for simultaneous sensing, monitoring, and treatment.

In short, MOF-based SERS sensors are moving toward a new generation of analytical tools that could significantly impact agricultural monitoring, clinical diagnostics, and environmental sensing. The next phase of research will likely focus less on incremental performance improvements and more on practical deployment, system integration, and reliability under real-world conditions. With continued effort in these areas, MOF–SERS platforms have the potential to become truly high-performance, field-ready sensors that extend far beyond the lab.

## Figures and Tables

**Table 2:** Comparison of MOF-based SERS sensors by material architecture, sensing mode, analyte, and analytical performance.

Type of Material	Analyte	Detection Method	Sample Matrix	LOD (μM)	Linear Range (μM)	Ref.
Ag@Fe_3_O_4_/UiO-66-NH_2_ (magnetic bulk substrate)	Cr(VI)	Magnetic MOF-assisted SERS	Water	0.078–0.089	-	[112]
Ag@ZIF-8@Au (rigid hybrid substrate)	Acetamiprid	MOF-protected plasmonic SERS	Soil, Lake water	9.03 × 10^−4^	10^−3^–10^2^	[113]
ZIF-67@AgNPs@PDA (rigid composite substrate)	Thiram (THR)	MOF-plasmonic SERS	Juice	3.2 × 10^−7^	-	[116]
MOF-imprinted paper strip (flexible paper-based sensor)	Thiacloprid	Paper-based MOF-SERS	Soil	0.04	-	[117]
Magnetic MOF ratiometric aptasensor (magnetic separation)	Patulin (PAT)	Ratiometric MOF-SERS	Juice	3.0 × 10^−4^	-	[118]

**Table 3:** Representative MOF-based SERS sensors for biomedical applications.

Type of Material	Analyte	Detection Method	Sample Matrix	LOD (μM)	Linear Range (μM)	Ref.
NiRs@MOF-74(Ni)/Ag	T-2 toxin	Magnetic aptamer SERS	Food samples	4.7 × 10^−4^	1.6 × 10^−3^–2.4	[126]
Au–Ag Janus@Au NPs	Deoxynivalenol	Magnetic aptamer SERS	Food samples	2.5 × 10^−4^	1.0 × 10^−3^–2.4	[127]
Mn/Fe-MIL(53)@AuNS–MBA	Shiga toxin II	SERS	Milk	1.5 × 10^−4^	9.1 × 10^−4^–1.8	[128]
Fe_3_O_4_@SiO_2_@Ag	Tacrolimus (FK506)	Magnetic sandwich SERS	Human blood	4.1 × 10^−4^	6.2 × 10^−4^–2.5 × 10^−2^	[132]
IRMOF-3@Au/PDMS	Histamine	Flexible aptamer SERS		3.3 × 10^−7^	9.0 × 10^−7^–3.6	[133]

## Abbreviations

4-MBA	4-Mercaptobenzoic acid
4-MP	4-Mercaptophenol
4-MPBA	4-Mercaptophenylboronic acid
4-NPH	4-Nitrophenylhydrazine
AgNP	Silver nanoparticle
AgNF	Silver nanoflower
Au NP	Gold nanoparticle
Au NS	Gold nanostar
Au@MOF	Gold nanoparticle encapsulated within MOF
AIE	Aggregation-induced emission
BMOF	Bimetallic metal–organic framework
CHA	Catalytic hairpin assembly
CRPC	Castration-resistant prostate cancer
CV	Crystal violet
DA	Dopamine
DFT	Density functional theory
EV	Extracellular vesicle
FP	Filter paper
HGSOC	High-grade serous ovarian cancer
hCE1	Human carboxylesterase 1
HPDTP-Al	Functionalized aluminum-based MOF (specific name)
I_1172_/I_1074_	SERS intensity ratio at 1172 cm^−1^ and 1074 cm^−1^
LDH	Layered double hydroxide
LSPR	Localized surface plasmon resonance
MOF	Metal–organic framework
Ln-MOF	Lanthanide MOF
mAuNP	Mesoporous gold nanoparticle
Ni-MOF	Nickel-based MOF
NP	Nanoparticle
PATP	p-Aminothiophenol
Pb^2+^	Lead ion
P-HKUST-1	Phosphonyl-functionalized HKUST-1
PVP	Polyvinylpyrrolidone
PSM	Post-synthetic modification
R6G	Rhodamine 6G
RSD	Relative standard deviation
RhB	Rhodamine B
SERS	Surface-enhanced Raman scattering
TB	Toluidine blue
Tet	Tetracycline hydrochloride
TENG	Triboelectric nanogenerator
UiO-66	University of Oslo-66 (Zr-based MOF)
UiO-66(NH_2_)	Amino-functionalized UiO-66
VOC	Volatile organic compound
ZIF	Zeolitic imidazolate framework
ZIF-8	Zinc-based zeolitic imidazolate framework-8
ZIF-67	Cobalt-based zeolitic imidazolate framework-67
